# Transferring climate change physical knowledge

**DOI:** 10.1073/pnas.2413503122

**Published:** 2025-04-08

**Authors:** Francesco Immorlano, Veronika Eyring, Thomas le Monnier de Gouville, Gabriele Accarino, Donatello Elia, Stephan Mandt, Giovanni Aloisio, Pierre Gentine

**Affiliations:** ^a^Centro Euro-Mediterraneo sui Cambiamenti Climatici Foundation — Euro-Mediterranean Center on Climate Change, Lecce 73100, Italy; ^b^Department of Computer Science, University of California, Irvine, CA 92697; ^c^Learning the Earth with AI and Physics, New York, NY 10027; ^d^Department of Earth System Model Evaluation and Analysis, Deutsches Zentrum für Luft- und Raumfahrt e.V., Institut für Physik der Atmosphäre, Oberpfaffenhofen, Wessling 82234, Germany; ^e^Department of Climate Modelling, University of Bremen, Institute of Environmental Physics, Bremen 28359, Germany; ^f^Department of Earth and Environmental Engineering, Columbia University, New York, NY 10027; ^g^Ecole Polytechnique, Palaiseau 91120, France; ^h^Department of Engineering for Innovation, University of Salento, Lecce 73100, Italy

**Keywords:** Machine Learning, temperature, CMIP6, projections, uncertainty

## Abstract

Precise and reliable climate projections are required for climate adaptation and mitigation, but Earth system models still exhibit great uncertainties. Several approaches have been developed to reduce the spread of climate projections and feedbacks, yet those methods cannot capture the nonlinear complexity inherent in the climate system. Using a Transfer Learning approach, we show that Machine Learning can be used to optimally leverage and merge the knowledge gained from global temperature maps simulated by Earth system models and observed in the historical period to reduce the spread of global surface air temperature fields projected in the 21st century. We reach an uncertainty reduction of more than 50% with respect to state-of-the-art approaches while giving evidence that our method provides improved regional temperature patterns together with narrower projections uncertainty, urgently required for climate adaptation.

## Significance

Earth system models are crucial tools for projecting global mean temperature rise based on various Shared Socioeconomic Pathways in the sixth phase of the Coupled Model Intercomparison Project. However, these models exhibit significant uncertainties that challenge governments and stakeholders in developing effective climate change adaptation strategies. This study demonstrates the use of Transfer Learning to constrain long-term projections of global temperature maps by efficiently combining models’ simulations with historical observations spatially resolved on a global scale. This allows to reduce the spread of multimodel mean temperature projections while enhancing the reliability of the associated regional patterns.

Climate change is affecting all aspects of the Earth system, impacting ecosystems’ health, placing new strains on infrastructures, and affecting human migration (1, 2). Earth system models are the main tools used for assessing our changing climate. These models project global mean temperature rise according to several Shared Socioeconomic Pathways (SSPs), which represent future socioeconomic development scenarios linked to societal actions, such as climate change mitigation, adaptation, and impacts (3).

However, Earth system models still exhibit substantial uncertainties in their projections, even for prescribed greenhouse gas concentrations, posing significant challenges for governments and stakeholders in developing climate change adaptation strategies. These uncertainties have not been reduced with the evolution of models and have even increased in the latest generation participating in the Coupled Model Intercomparison Project Phase 6 (CMIP6) (4–6). For instance, the transient climate response—i.e., the surface temperature warming at the time of carbon dioxide (CO_2_) doubling in response to a yearly 1% increase in per-year CO_2_ concentration—produced by CMIP6 simulations is larger than the one produced by CMIP3 and 5 models ensembles (7). In CMIP6, the equilibrium climate sensitivity, i.e., the global temperature increase at equilibrium for a doubling of CO_2_, was the largest of any generation of models since the 1990s, ranging from 1.8 to 5.6 °C (7). It is well known that the majority of uncertainties in climate projections can be attributed to small-scale and “fast” physical processes, including but not limited to clouds, convection, and ocean turbulence (6, 8–10). By better constraining these physical processes, which are observable on a day-to-day basis, it would be possible to reduce the associated uncertainties.

Some of those issues are reflected in the inconsistency of CMIP6 models to reconstruct temperatures observed in the past (11). The models’ parameters calibration can be challenging due to data, time, and computational limitations (12). This calibration problem—together with errors arising from model structural assumptions, scenario uncertainty, and internal variability (13)—hampers the development of models that are fully aligned with historical observations (12), raising questions about the reliability of subsequent climate projections (14).

Several studies have attempted to constrain CMIP6 simulations with observational data by employing a variety of techniques (e.g., paleoclimate reconstructions, emergent constraints, model weighting, etc.). One common approach is the use of Reduced-Complexity Models (RCMs), also referred to as emulators. These are simplified physics-based models designed to replicate the large-scale response of Earth system models at reduced computational cost. Their parameters can be easily calibrated under reasonable priors (often informed by Earth systems models’ distributions) to produce historically consistent hindcasts, a critical condition for trust in future projections (14–16). However, RCMs usually do not capture the spatial details or accuracy required for detailed climate projections (16).

In the present study, we demonstrate that Transfer Learning (TL), a recent branch of Machine Learning (ML), can be utilized to efficiently leverage knowledge from an ensemble of CMIP6 Earth system models and constrain it to match historical observational data. TL enables the exploitation of knowledge acquired by a pretrained model on a data-rich task as a foundation for enhancing performance on a new but related task within the same domain, even with limited data availability (17). We show that using this approach, the uncertainty associated with multimodel temperature projections can be reduced by optimally fusing models projections and historical observations while resolving the regional patterns of climate change. This helps enhance the representation of future projections and their associated spatial patterns, particularly over time scales of a few details which are critical for policymakers (14).

## Constraining Climate Projections

Various approaches have been proposed to reduce the uncertainties of climate models projections. They leverage current or past climate observations to refine climate sensitivity estimates (18, 19).

One group of approaches has been exploiting paleoclimate proxies (i.e., surrogates for climate variables, such as temperature), especially chemical tracers that are now routinely simulated in Earth system models, to reduce and better constrain the range of climate sensitivity (20). Paleoclimate records offer tremendous potential, but paleoclimate proxies are not exempt from potential issues since they are only surrogates of the actual variable of interest, and sometimes strong assumptions might be required to link those proxies to climate variables.

A second group of approaches has used more recent climate observations—such as those from the 20th century which do not require proxies but cover a shorter time period—to constrain the range of climate sensitivity. One of these methods is the use of emergent constraints. They relate a physical process, which is an important regulator of climate sensitivity (e.g., low cloud reflectivity), and its spread across models to an observation that is used to constrain future climate sensitivity within a Bayesian framework (10, 21–24). These techniques, however, also suffer from several issues as they assume a linear relationship between the constraining and the target variable, while many important climate feedbacks are nonlinear (24–27). Emergent constraints are typically cast in terms of a univariate constraint, whereas many processes can interact and be multivariate. Moreover, these constraints are critically dependent on the models ensemble used (28) and do not account for the pattern effect, which refers to the dependence of the Earth’s outgoing radiation on the global surface warming pattern and is important for climate sensitivity (29).

Simple toy zero-order models of the Earth’s climate can also be used to understand the response of the global climate (30, 31) and especially the role of different climate feedbacks, such as those from water vapor or clouds. Recently, also RCMs have been developed with this aim, resulting less computationally demanding and representing the global climate at annual scales in terms of macroproperties of the climate system. They allow to investigate uncertainties across various components of the climate system and provide a framework to perform probabilistic calibrations of their parameters based on historical observations and various lines of evidence (15, 16). In a recent work, Smith et al. (14) calibrated the FaIRv2.1.0 model with emissions and observational constraints updated through 2022 to provide near- and long-term warming projections (fair-calibrate v1.4.1). Their study also includes an updated calibration of FaIR that was previously developed in the context of the Sixth Assessment Report of the Intergovernmental Panel on Climate Change (IPCC AR6) which uses historical emissions data up to 2014 and projections thereafter (fair-calibrate v1.4.0). Meinshausen et al. (32) used the probabilistic emulator MAGICC7 (33) to conduct a comprehensive evaluation of long-term temperature projections according to the 2030 nationally determined contributions and long-term low-emission development strategies submitted by several countries around the globe. Quilcaille et al. (34) integrated OSCAR v3.1—an emulator built as a combination of modules, each dedicated to different components of the Earth system that can be calibrated separately—with historical temperatures and forcing constraints. Yet, the spatial patterns of climate response and sea surface temperature or the subtle response of cloud-circulation feedback are important for the overall climate response. These subtleties cannot directly be resolved if RCMs are used (30, 35).

More accurate projections can also be achieved by applying optimal corrections to Earth system models based on historical observations. Indeed, available observed warming trends over the last decades have been used in several studies to constrain model-based temperature projections over the 21st century. Tokarska et al. (36) reduced the uncertainty in future projections by downweighing those CMIP6 models whose simulation results are not in line with historical warming. Ribes et al. (37) constrained global mean temperature projections using an adaptation of Gaussian process regression (also known as kriging) combining CMIP6 simulations and historical warming observations since 1850. Liang et al. (38) exploited a weighting method that takes both model quality and independence into account (39) to give more weight to CMIP6 models that better match the observed 1970 to 2014 warming. It is worth noting that these constraints do not consider the pattern effect in their temperature projections as they are computed against global average temperatures.

Finally, the IPCC Working Group 1 (WG1) assessed the global surface air temperature change in the AR6 using multiple lines of evidence, including CMIP6 projections up to 2100. CMIP6 projections were combined with observational constraints on simulated past warming to update estimates in the AR6 (40).

Recently, TL has proven to be a powerful tool in scientific applications such as weather/climate prediction (41) and environmental remote sensing (42). TL techniques have been successfully applied to merge the knowledge of climate models simulations and observations to make long-lead El-Niño Southern Oscillation forecasts (43, 44). In general, there has been a growing interest in the scientific community to employ ML to improve climate models projections, for instance, by enhancing parameterizations. There have been some initial attempts to build ML-based climate emulators as well. Examples are, for instance, the AI2 Climate Emulator (45)—which is trained to reproduce a physics-based atmospheric model and predicts several diagnostics; Weber et al. (46) who investigate the use of Deep Neural Networks (DNNs) as emulators to produce short-term precipitation forecasts; or ClimaX, a foundation model trained on CMIP6-derived datasets that can be employed for both weather and climate-related downstream tasks (47). However, with respect to these approaches, this work represents application of ML and especially TL to simultaneously reduce the spread of global climate temperature projections and improve the corresponding regional patterns.

## Results and Discussion

### Leave-One-Out Cross-Validation Approach.

This work aims to learn, i.e., acquire knowledge, from historical and projected climate simulations from CMIP6 models constrained by historical observations to provide more precise and reliable climate projections. This learning is first acquired by pretraining 66 DNNs, each dedicated to one of 22 CMIP6 models across three SSP scenarios: SSP2-4.5, SSP3-7.0, and SSP5-8.5. In this initial phase, each DNN learns the complex relationships between CO_2_ equivalent forcing and CMIP6 temperature at a regional scale, capturing the diversity of different models’ responses. Given the lack of observations, in the future, validating this approach is essential before integrating historical observations. Therefore, a rigorous testing phase is performed using a leave-one-out cross-validation (also known as model-as-truth) strategy (48) where CMIP6 models are used as “synthetic observations.” This provides a systematic assessment of the DNNs’ ability to generalize and adjust projections across different CMIP6 simulations, adding robustness and confidence to the approach (see *Materials and Methods* for further details).

In the following, we use SSP2-4.5 as a reference since low-emission scenarios are currently more likely by the end of the century than the high-emission SSP5-8.5 (49). The global average temperature error, rms error (RMSE), percentage of uncertainty reduction, and accuracy, along with 5 and 95% in 2081 to 2098, are computed for the three SSP scenarios considered (*SI Appendix*, Table S2 and *Materials and Methods*).

**Fig. 1.**
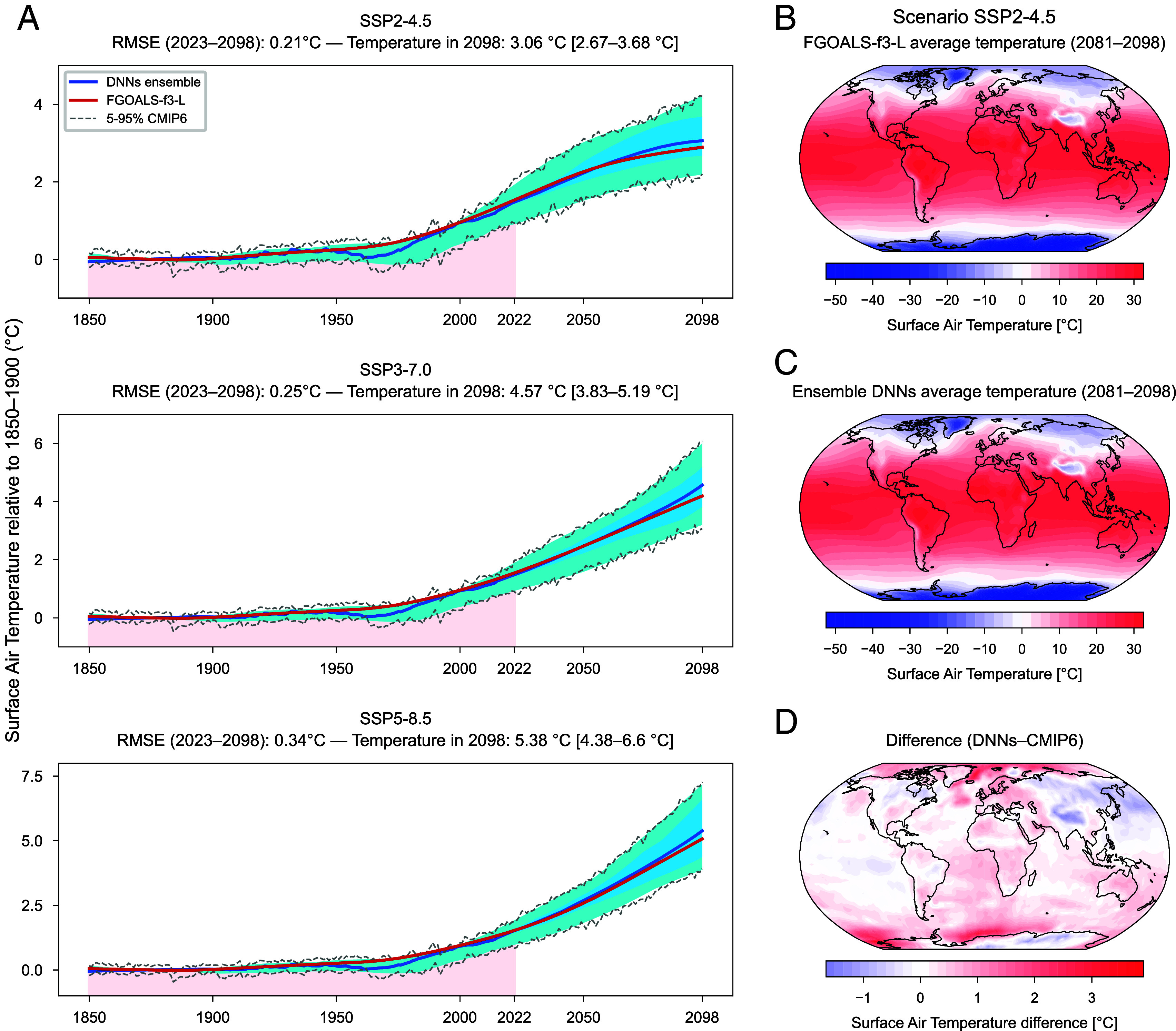
Leave-one-out cross-validation example (here for FGOALS-f3-L) for the three SSPs considered in the study. (*A*) Global average warming (baseline: 1850 to 1900) projected by the DNNs ensemble (average across DNNs; bold blue line) for each SSP scenario and FGOALS-f3-L simulation data (bold red line). The projections are generated after TL each DNN on the FGOALS-f3-L historical simulations. Pink shadings show the training set (1850 to 2022). The 5 to 95% ranges are reported for the DNNs (dark blue shading; numerical values for the 5 to 95% range of warming prediction in 2098 are present in square brackets), the smoothed CMIP6 simulations (light blue shading), and the original CMIP6 simulations (dashed gray lines). (*B*–*D*) Maps of surface air temperature projected in 2081–2098 by FGOALS-f3-L (*B*) and by the DNNs ensemble (*C*) under SSP2-4.5 scenario. (*D*) The difference between the DNNs ensemble and CMIP6 ensemble temperature maps is also reported.

The leave-one-out cross-validation shows a mean global average error of 0.28 °C and a mean global average RMSE of 0.29 °C, in the 2081 to 2098 time period, with respect to the synthetic observations across all the 22 taken-out models under SSP2-4.5 (*SI Appendix*, Table S2). The description of each metric is reported in Materials and Methods (section Metrics). As an example, Fig. 1*A* shows the narrow 5 to 95% confidence range (2.67 to 3.68 °C) of the global average warming for 2098 relative to the 1850 to 1900 base period, when FGOALS-f3-L is used as synthetic observation. This reveals that the proposed approach is effective at narrowing the temperature uncertainty range (i.e., increasing the precision). Moreover, the global average error between the average temperatures projected by the DNNs ensemble (average across DNNs, bold blue line in Fig. 1*A*) and the synthetic observations from FGOALS-f3-L (bold red line in Fig. 1*A*) is equal to 0.18 in the 2081 to 2098 time period, as reported in *SI Appendix*, Table S2. This confirms that good accuracy is also achieved. During the leave-one-out cross-validation, the role of TL is to transfer prior information from the CMIP6 models and combine it with the historical simulation of the taken-out model (1850 to 2022), thus enabling the DNNs to accurately extrapolate temperatures in the future period. In addition, the fine-tuned DNNs are also able to spatially project all the complexity of surface air temperature consistently replicating the details of future regional features—such as the land-ocean contrast, the Arctic Amplification, the gradient of warming between Tropics and mid-latitudes, or colder temperatures over Greenland (Fig. 1 *B*–*D*).

### TL on Observational Data.

The leave-one-out cross-validation procedure represents a proof of concept to demonstrate the effectiveness of transferring knowledge from the climate models to synthetic observations, allowing extrapolation beyond the historical regime. The same strategy is ultimately applied to real observed historical temperature data, which serves as a constraint to refine the knowledge initially gained from CMIP6 simulations and align the DNNs emulators with real-world temperatures and their trends. As a result, the fine-tuned DNNs aim to provide more reliable temperature projections for future scenarios by leveraging both simulated and observed data (see *Materials and Methods* for further details).

**Fig. 2.**
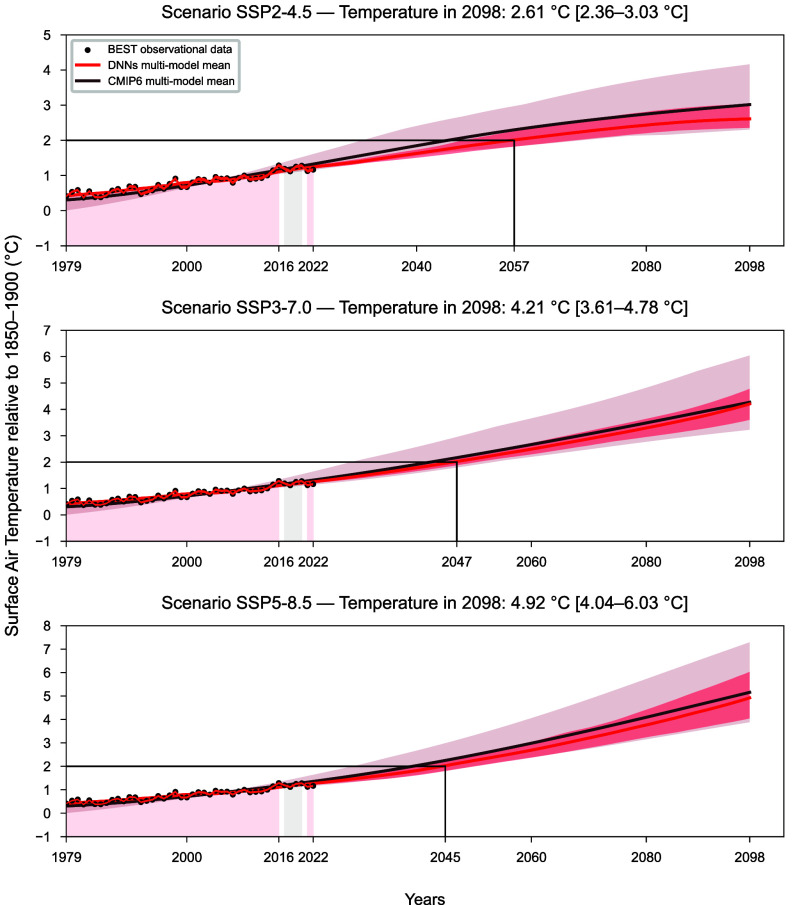
TL on observations. DNNs ensemble projections (average across DNNs, bold red line) of global average warming relative to 1850 to 1900 for each scenario. The projections are generated after TL (training set, pink shading: 1979 to 2016, 2021, 2022; validation set, gray shading: 2017 to 2020) each DNN on BEST historical observational data (black dots). Each plot also shows the year the 2 °C Paris Agreement threshold will be reached according to the DNNs ensemble projections. The 5 to 95% ranges of the projections produced by the DNNs (red shading) and the unconstrained smoothed CMIP6 simulations (brown shading) are reported. The unconstrained CMIP6 ensemble simulation (average across models, bold brown line) is shown as well. For each plot, numerical values of the 5 to 95% range of warming predictions in 2098 are present in square brackets.

**Fig. 3.**
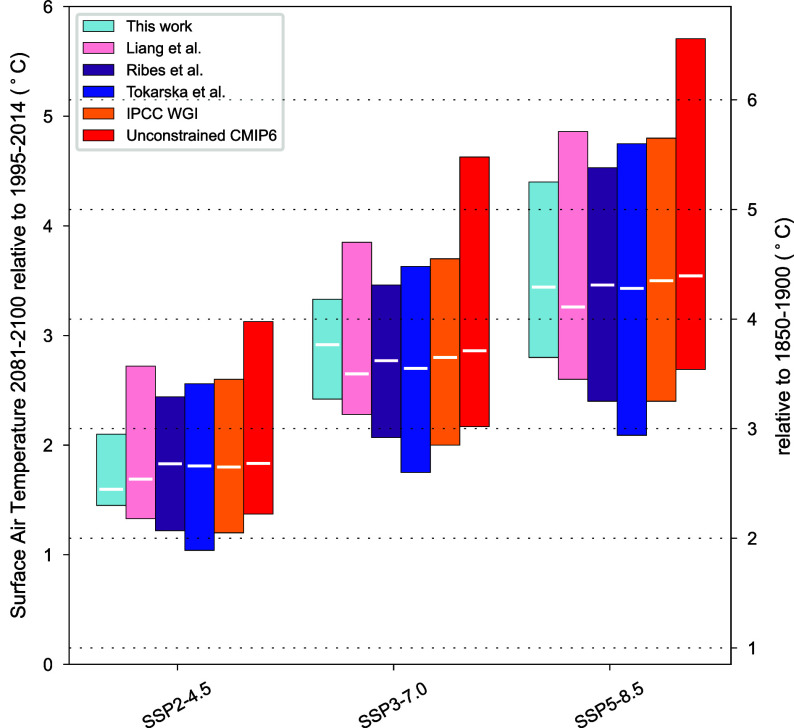
Global surface air temperature changes for the long-term period (2081 to 2100). Global 5 to 95% warming ranges for the long-term period (2081 to 2100) relative to 1995 to 2014 (*Left y* axis) and 1850 to 1900 (*Right y* axis) for SSP2-4.5, 3-7.0, and 5-8.5 scenarios. White lines for each box plot represent the temporally averaged median values. Note that the bar plots for Ribes et al. and this work are computed in the 2081 to 2098 time period. The remaining ones are computed in the 2081 to 2100 time period. These results extend those reported in Chapter 4 of the IPCC AR6 (40).

In the following, the SSP2-4.5 scenario is used again as a reference and predicted future warming values are relative to the 1850 to 1900 baseline period. The ensemble mean and spread (5 to 95% range) across the DNNs are used to project future climate change. Our estimated global annual mean temperature increase by 2098 is 2.61 °C (2.36 to 3.03 °C). This can be compared to the CMIP6 intermodel equal-weight mean of 2.98 °C (2.28 to 4.13 °C) (Fig. 2). The fine-tuned DNNs project lower temperatures compared to the warmest CMIP6 models whose warming rates might be unrealistically too high according to several lines of evidence (50). Concerning the 2081 to 2098 time period, we observe a reduction of about 63% in the overall uncertainty range compared to the unconstrained CMIP6 models (Fig. 3 and *SI Appendix*, Table S3). It is worth noting that the spread in the CMIP6 global mean temperature projections is typically sensitive to the subset of models used for the ensemble in the standard CMIP6 projections. This is not the case in our approach, as all the DNNs trained on independent models and then fine-tuned on historical temperature data are projecting nearly the same global temperature rise after TL (Fig. 2). Further, model filiation does not impact the result, as the models exhibit the same performance whether or not they share some lineage (*SI Appendix*).

In comparison to other state-of-the-art methods, including some RCMs, aimed at narrowing down the model-based projections uncertainty, we find a 47% reduction in projections uncertainty with respect to Ribes et al. (37), 53% with respect to Liang et al. (38), and 57% with respect to Tokarska et al. (36) under SSP2-4.5. Moreover, we obtained a 54% reduction with respect to the 5 to 95% range assessed by IPCC WG1 AR6 (40) and about 60% compared to the estimate provided by both fair-calibrate v1.4.0 and v1.4.1 (14) (Fig. 3 and *SI Appendix*, Table S3). Even our near-term (2021 to 2040) and mid-term (2041 to 2060) projections result in an agreement but with a smaller spread with respect to IPCC WG1 AR6 evaluation, fair-calibrate v1.4.0, and fair-calibrate v1.4.1 (*SI Appendix*, Table S4).

We also compared our results with the estimates provided by two additional calibrated RCMs. For OSCAR v3.1 (34), the authors report means and SD in 2041 to 2050 and 2091 to 2100 that we computed and compared in *SI Appendix*, Table S5. Overall, we observe comparable values between our results and the constrained estimates of OSCAR v3.1, except for the projection in 2091 to 2100 under SSP5-8.5 exhibiting higher temperature value and SD projected by the DNNs ensemble. Regarding MAGICC7 (32), the authors examine the implications for the long-term temperature increase resulting from the 2030 nationally determined contributions and current energy policies. They identify eight emission levels and rates of change broadly similar to SSP2.4.5, in addition to two other scenarios that include long-term low-emission development strategies as well. For our comparison, we focused on the eight scenarios that are closer to SSP2-4.5 and selected the one with the narrowest uncertainty range. The authors report a 5 to 95% temperature range of 1.59 to 3.31 °C by 2100 relative to 1850 to 1900. Considering this estimate, our projection for 2098 under SSP2-4.5 (Fig. 2) exhibits a 61% reduction in the uncertainty range.

The aforementioned evaluations are also confirmed for SSP3-7.0 and 5-8.5 (Fig. 3 and *SI Appendix*, Tables S3 and S4).

The Paris Agreement aims to “hold the increase in the global average temperature to well below 2 °C above preindustrial levels and to pursue efforts to limit the temperature increase to 1.5 °C above preindustrial levels” (40). From the analysis made by the IPCC WG1 in the AR6, the central estimate of crossing the 1.5 °C threshold is found to be in the “early 2030s” (for all SSPs except 5-8.5), about 10 y earlier than the midpoint of the likely range (2030 to 2052) communicated in the Special Report on global warming of 1.5 °C (51) in which continuation on the current warming rate was assumed (40). Moreover, surpassing the 1.5 °C threshold was recently estimated by the European Center for Medium-Range Weather Forecast between 2030 and 2035, using a linear extrapolation of the current global warming trend (52).

Diffenbaugh and Barnes (53) predicted that 1.5 and 2 °C will be reached in 2033 (2028 to 2039) and 2049 (2043 to 2055), respectively, under SSP2-4.5. According to our results, the 1.5 °C global threshold (relative to 1850 to 1900) will be exceeded in 2035 (2031 to 2040). Similarly, the 2 °C threshold will be exceeded in 2057 (2049 to 2068) (*SI Appendix*, Table S6). Each of those years is computed as the first year at which 21 y running averages of surface air temperature exceed the given global warming level, as done in Chapter 4 of IPCC WG1 AR6 (40).

### Structural and Parametric Errors.

Two natural questions come to mind after demonstrating the performance of the DNNs. First, why can the DNNs project climate change so well? And, second, is not the historical data used twice given that some of them are used during the model tuning? Those two questions boil down to the same underlying causes. Earth system models are a simplified representation of the complex physical, chemical, and biological processes of the real world. As such, they inherently make assumptions regarding the representation of the processes in terms of the equations and their structure (e.g., the complexity), as well as the values of parameters used in those equations. Some of the available historical data are used to tune the major models’ parameters (e.g., cloud entrainment rate or microphysical parameters) to match the historical climatology or some modes of climate variability, such as El Niño (54, 55). Yet, each model is inherently limited by its structural assumptions and thus cannot optimally use existing data as it can only work within a subspace restricted by its complexity and inherent structure. Our DNNs, instead, learn how to best leverage both (structurally deficient) physics of climate simulations and historical data to improve the projections of regional temperature, strongly reducing some of the temperature biases that characterize most Earth system models.

**Fig. 4.**
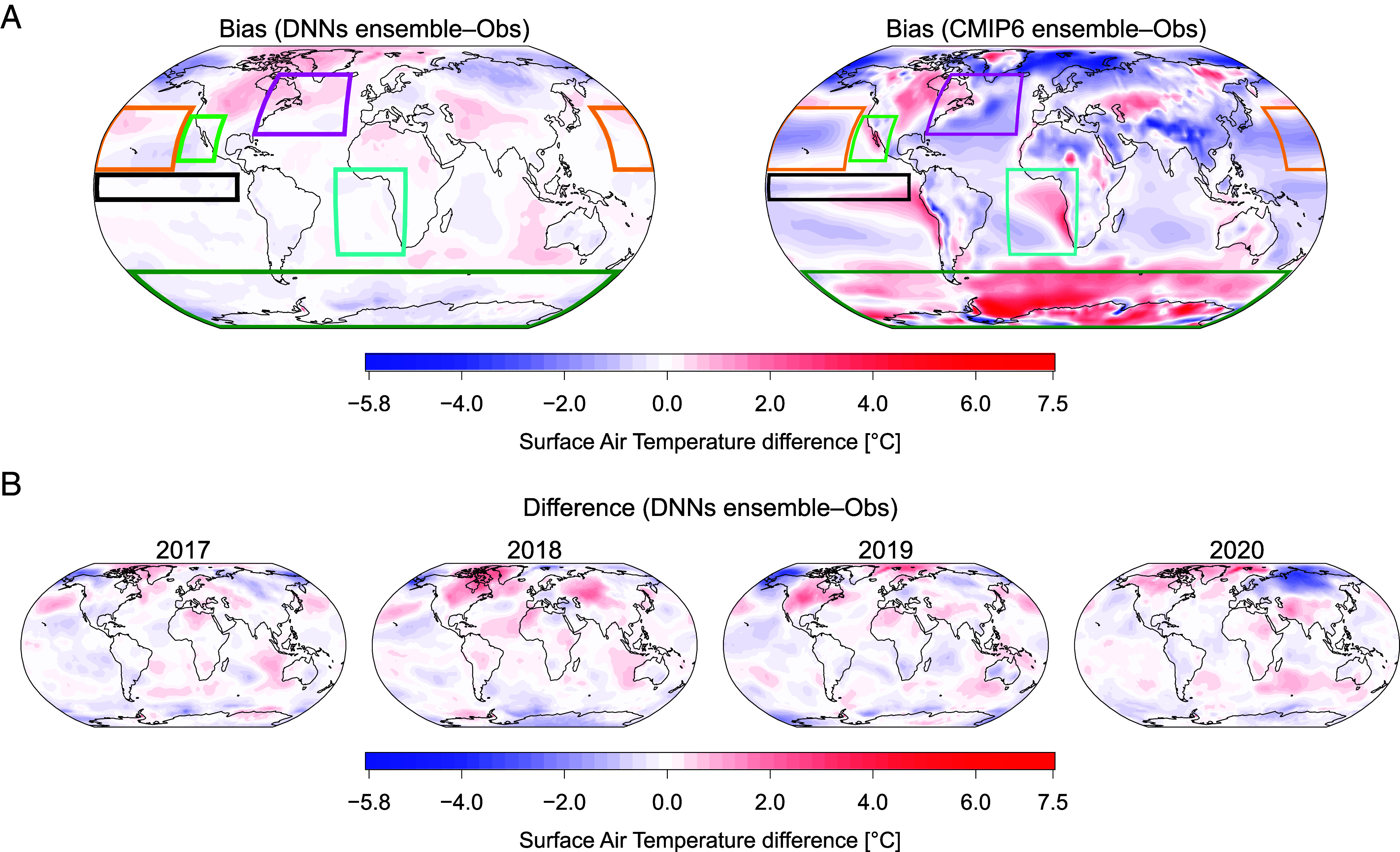
Historical bias surface air temperature maps. (*A*) Average bias surface air temperature maps in validation years (2017 to 2020) of both DNNs and unconstrained CMIP ensembles (average across models) for SSP2-4.5. Some well-known biases are selected and highlighted with the colored boxes—Antarctic (green), cold tongue (black), Gulf Stream (purple), South East Atlantic (light blue), North West Pacific (orange), and North East Pacific (light green). The bias maps are computed by averaging over time the temperature maps generated by the DNNs and CMIP6 ensembles and subtracting the observation maps averaged over the same years. (*B*) The difference in the surface air temperature maps of the DNNs ensemble with respect to the observation data in each single year is also reported.

One of the major biases is the “cold tongue” and its extension along the equatorial band, which is typically too cold by about 2 °C (56) and present in all three generations of CMIP models (57). The DNNs ensemble improves the cold tongue bias by predicting higher surface air temperature values than the CMIP6 ensemble in the historical period (Fig. 4). Another bias typically present in climate models concerns the Arctic Amplification (58, 59). It has been shown that, from 1979 to 2021, the Arctic warmed nearly four times faster, and both CMIP5 and CMIP6 models underestimate it (58). The maximum warming is observed in the Eurasian sector of the Arctic Ocean, near Svalbard and Novaya Zemlya (58). This pattern is captured and improved by the DNNs ensemble after the inclusion of the observational constraint and is exploited to predict temperature regional variations (Fig. 4). Furthermore, coupled Earth system models are affected by sea surface temperature biases in the location and structure of the Gulf Stream (60, 61). In particular, warmer temperatures are simulated in the North Atlantic region centered on the Mid-Atlantic bight, where the modeled Gulf Stream separates from the coast further north than observations (62, 63). Also, a well-known and long-standing issue in ocean modeling is the cold bias located to the east of the Grand Banks of Newfoundland (62), where the Gulf Stream ends and the North Atlantic Current begins; however, in higher-resolution models, this representation is improved (60, 64, 65). Our DNNs improved it as well, generating lower surface air temperatures in the aforementioned region (Fig. 4).

High sea surface temperatures in the western Pacific warm pool and lower temperatures in the eastern Pacific cold tongue create a zonal contrast in the tropical Pacific atmosphere-ocean state (66) which can diverge across future projections (65). Most CMIP models project a higher warming in the equatorial central-eastern Pacific than the western Pacific, which corresponds to a weakening of the temperature gradient, often called an “El Niño-like” warming pattern (66–72). Yet, this appears to be opposite to the strengthening observed since the mid-twentieth century, which appears to be a “La Niña-like” warming (66, 67, 71). We acknowledge that determining future responses from unforced natural multidecadal variability or from a forced response over short periods of time is not trivial (70, 71, 73). Nonetheless, the contribution of natural variability to multidecadal trends appears relatively small in this region. Thus this suggests a systematic model bias in response to anthropogenic forcing (70, 73) as observations are outside the models’ range (67). Moreover, it has been shown that a physically consistent response to warming could be La Niña-like and that it could have been detectable since the late twentieth century (71), which is aligned with our results (*SI Appendix*, Figs. S9 and S10).

## Conclusions

This work demonstrates that DNNs initially trained to emulate Earth system models and then fine-tuned using historical global surface air temperature maps can project climate change for prescribed greenhouse gas concentrations with reduced uncertainty and improved regional temperature patterns.

Using this strategy, we substantially reduced the 5 to 95% range of projected global surface air temperature across SSP2-4.5, 3-7.0, and 5-8.5 scenarios. Specifically, concerning the 5 to 95% warming confidence range in 2081–2098 under SSP2-4.5, we obtained a reduction of 47% with respect to the best state-of-the-art approach (37) and 54% compared to the IPCC WG1 AR6 (40). An improvement with respect to other methods was also observed under SSPs 3-7.0 and 5-8.5. Our end-of-century estimate of global surface air temperature increase (relative to 1850 to 1900) is 2.61 °C (2.36 to 3.03 °C) for SSP2-4.5, which translates into exceeding the 1.5 °C threshold of the Paris Agreement in 2035 (2031 to 2040) under SSP2-4.5. Under the same scenario, the 2 °C threshold will be exceeded in 2057 (2049 to 2068). Our results are in line with recent estimates from the state-of-the-art methods [including IPCC WG1 AR6 (40)] and CMIP6 Earth systems models but with reduced uncertainty.

In addition, a significant aspect of our work is the projection of annual surface air temperature maps with global coverage, as opposed to only providing globally averaged annual values. The regional projections produced by the DNNs ensemble show improved regional patterns compared to CMIP6 models. It is important to note that while our findings indicate that the TL approach effectively improves well-known temperature biases exhibited by CMIP6 models in the historical period, this does not necessarily imply a correction of these biases in future projections. This is due to the lack of direct observational data of unknown future responses. Indeed, substantial uncertainties still affect future greenhouse gas concentration scenarios, especially for end-of-century projections. Some of those uncertainties relate to projections of the ocean and terrestrial carbon uptake (74, 75), even though there have been recent attempts to refine those model estimates (76). Yet, reducing greenhouse gas emissions is clearly the only path forward to meet the limits set by the Paris Agreement.

Some other questions related to the results achieved in this work remain open and deserve further investigation. For instance, the inclusion of interannual variability would be essential to characterize extreme events and is left for future work. Exploring a hybrid approach where DNNs are applied to bias-corrected CMIP6 simulations would be a further avenue of research, with the aim of potentially enhancing the reliability of our projections, yet potentially at the expense of explainability.

Furthermore, the dominant drivers of forced climate change on global and regional scales have been both greenhouse gases and anthropogenic aerosols since the Industrial Revolution (77, 78). These two factors differ not only in their global mean radiative forcing impacts but also in their spatial and temporal evolutions. Indeed, long-lived greenhouse gases are globally well-mixed and have increased monotonically over the past decades. In contrast, anthropogenic aerosols are geographically inhomogeneous due to their short atmospheric residence time. Different regions of the world exhibited contrasting levels of aerosol emissions in the past, which even changed over time with complex spatial patterns and time evolutions. These distinct forcing characteristics present a challenge to the study of regional and global climate response, even if capturing the long-term aerosol trends is crucial to provide reliable temperature estimates and projections (79–81). This deserves future research despite the challenges posed by the lack of good constraints on the spatial variability of historical aerosol concentrations.

Another important consideration in our approach is that the same forcing is used for all 22 CMIP6 models under a specific SSP scenario. This implies that the uncertainty in historical aerosol forcing is represented by the spread among CMIP6 models. In IPCC AR5 (82), it was highlighted that the spread of aerosol forcing among CMIP5 models was narrower than the full range derived from observations and other lines of evidence, indicating that model-based uncertainty estimates may not fully capture the entire variability. Although AR6 provides updated effective radiative forcing (ERF) estimates for aerosols, substantial uncertainties still remain (83–85). This limitation suggests that our method, while improving projections by constraining simulations to observational temperature data, may still underestimate the full range of historical aerosol forcing uncertainty. Future investigations will explore ways to comprehensively integrate observational constraints on aerosol forcing to better account for these uncertainties.

Nevertheless, this work provides evidence of the efficacy of ML in optimally integrating historical observations and climate models knowledge, suggesting the potential for improved models’ precision and reliability in climate projections and a strengthened foundation for future predictions.

## Materials and Methods

### Earth System Models.

We use global surface air temperature maps simulated from 1850 to 2098 by 22 CMIP6 Earth system models (*SI Appendix*, Table S1) under SSPs 2-4.5, 3-7.0, and 5-8.5. For each model and scenario, we employ a single ensemble member. Specifically, the r1i1p1f1 member is chosen as it is frequently the primary member in CMIP6 models and is also used in IPCC WG1 AR6 (40) for evaluating temperature projections. However, this member was unavailable for CNRM-CM6-1, CNRM-ESM2-1, and UKESM1-0-LL. For these models, we opted for r1i1p1f2. Selecting a single member per model helps us manage computational complexity within our framework while ensuring alignment with the IPCC’s methodology (40).

Furthermore, some of the CMIP6 models simulations are available at a spatial resolution of about 250 km and others at 100 km. The conservative remapping (86) is employed to align all simulations with the CanESM5-CanOE grid, which is the lowest-resolution one among all those available, with 64 × 128 grid points. The coarsest spatial resolution is selected to avoid any synthetic information that would be added in case of remapping to a higher-resolution grid. The CMIP6 simulated maps are gathered at a monthly temporal resolution and subsequently averaged over a year to generate the corresponding annual version, which is aligned with the temporal resolution (annual) of CO_2_ equivalent input data.

### CO_2_ Equivalent Data.

A single annual CO_2_ equivalent value is used as predictor for each DNN. These CO_2_ equivalent values are computed from ERF estimates which take into account aerosols and greenhouse gases (e.g., CO_2_, methane, nitrous dioxide, etc.) and are simulated by the Minimal CMIP Emulator v1.2 RCM (87). We have one ERF value per year per SSP scenario. For each ERF value, we iteratively calculate the corresponding CO_2_ equivalent value such that, when entered into a CO_2_ radiative forcing formula, it produces an output within a tolerance of less than 1e-5 compared to the ERF value. This calculation results in three time series of CO_2_ equivalent values from 1850 to 2098, one for each SSP scenario, with one CO_2_ equivalent value per year. This is used as a single input for the DNNs throughout the pretraining, leave-one-out cross-validation, and TL on observations phases. The CO_2_ radiative forcing formula used in this work is reported below. It was introduced by Meinshausen et al. (88) to represent radiative forcing after stratospheric adjustments, relative to preindustrial (1750) levels, and is an optimized modification of the simplified formula presented by Etminan et al. (89).

$$RF_{CO2}=\left(\alpha \prime +\alpha_{N_{2}O}\right)\cdot ln\left(\frac{C}{\mathrm{Co}}\right)$$

where:

$$C_{\alpha_{\mathrm{MAX}}}=C_{0}-\frac{b_{1}}{2a_{1}}\approx 1808ppm$$

$\alpha \prime =d_{1}-\frac{b_{1}^{2}}{4a_{1}}$ for $C>>>C_{\alpha_{\max}}$

$\alpha \prime =d_{1}+a_{1}{\left(C-C_{0}\right)}^{2}+b_{1}\left(C-C_{0}\right)$*, for* $C_{0}< <C< <C_{\alpha_{\max}}$

$\alpha \prime =d_{1}$* , for* $C< <C_{0}$

$$\alpha_{N_{2}O}=c_{1}\cdot \sqrt{N}$$

$$a_{1}=-2.4785\times 10^{-7}Wm^{-2}ppm^{-1}$$

$$b_{1}=0.00075906Wm^{-2}ppm^{-1}$$

$$c_{1}=-0.0021492Wm^{-2}ppb^{-0.5}$$

$$d_{1}=5.2488Wm^{-2}$$

$$C_{0}=277.15ppm$$

### BEST Observational Data.

We use historical surface air temperature estimates from the global Berkeley Earth Surface Temperatures (BEST) (90) gridded data, which are provided on a 1° × 1° latitude/longitude grid with a monthly temporal resolution and gathered from 1850 to 2022. Specifically, we select the BEST maps with air temperatures at sea ice, in which temperatures in the presence of sea ice are extrapolated from land-surface air temperature. This revealed to be a more sensible approach for capturing climate change, especially at the poles. Indeed, the change of air temperatures over sea ice can be large even if the sea surface temperature under sea ice is not changing, since the latter is strictly connected to the water freezing point and can only vary with changes in sea ice cover. Over the last decades, the Arctic region was characterized by a very strong warming trend during the winter season, and this translated into an additional ~0.1 °C global-average temperature rise during the 19th century with respect to estimates not including such changes (i.e., estimates based on sea surface temperature under sea ice) (90).

The conservative remapping (86) is used to align the BEST data to the same CanESM5-CanOE grid used for CMIP6 data, thus generating temperature fields of size 64 × 128 and averaged over time to obtain a single map per year. Although the temporal coverage of the BEST dataset starts from 1850, maps prior to 1979 are excluded after the remapping process due to the lack of data in many regions at the time and thus reduced accuracy. For this reason, the temporal domain used is 1979 to 2022.

In order to account for aleatoric uncertainty (i.e., uncertainty related to the data’s inherent randomness and stochasticity), a noise is added to each annual BEST map by sampling the values from a Gaussian distribution (91, 92) with 0 mean and SD equal to the annual uncertainties—provided by the Berkeley Earth group and available with the dataset. These uncertainties represent the statistical and spatial undersampling effects as well as ocean biases (90). To include epistemic uncertainty (i.e., uncertainty due to the model’s lack of knowledge about the phenomenon of interest), an ensemble technique (93) is exploited. Specifically, five datasets are built for each CMIP6 model and for each SSP scenario by sampling and adding the random Gaussian noise to the BEST temperature maps, thus obtaining an ensemble of 330 (i.e., 5 × 22 × 3) datasets of historical observations. This allows estimating structural and aleatoric uncertainties and the noise due to internal climate variability. We tried 10 and 20 BEST-perturbed datasets per model and scenario as well but did not obtain substantial improvements. We did not evaluate Monte Carlo dropout (94) for the quantification of aleatoric uncertainty as it has been shown to underestimate the uncertainty (95–97).

### TL Approach.

This work introduces a TL framework to improve global surface air temperature projections by leveraging DNNs pretrained on CMIP6 simulations and fine-tuned on observational data. The approach involves training DNNs to emulate the spatial temperature patterns of climate models and then refining them using historical observations, and can be viewed as a middle ground between purely model-based and purely data-driven projections. This strategy aims to reduce uncertainty in multimodel projections by blending simulated and real-world data, validated through a cross-validation-like process.

The first step of the algorithm involves the use of 66 DNNs to emulate the global annual surface air temperature maps simulated by 22 CMIP6 models (*SI Appendix*, Table S1) under SSPs 2-4.5, 3-7.0, and 5-8.5. An individual DNN is trained for each CMIP6 model simulation (*SI Appendix*, Fig. S1*A*). Each DNN predicts a single temperature map per year starting from the corresponding annual CO_2_ equivalent concentration. In total, 66 DNNs are implemented and pretrained, representing the combination of 22 CMIP6 models and 3 SSP scenarios. The pretraining is performed using data from 1850 to 2098, since 2098 is the last projection year available in all the selected CMIP6 simulations. Moreover, the years from 2070 to 2080 are reserved for validation purposes. The primary goal of each DNN in this pretraining phase is to replicate the CMIP6 simulation it is trained on as closely as possible, effectively building a robust, tunable emulation of CMIP6 temperature projections and capturing the link between CO_2_ equivalent values and temperature spatial patterns (which are inherently complex due to the diversity of responses across regions and scenarios).

This work proposes the use of TL to combine the models’ simulations with the information from historical observational data with the ultimate goal of reducing the uncertainty of multimodel projections. To identify the right amount of information transfer and assess the degree of uncertainty reduction and model fit, we proceed as follows. For each scenario, one of the 22 CMIP6 simulations is taken out and used as ground truth for validation in a leave-one-out cross-validation framework (48). This approach allows a robust testing of the TL phase by assessing each DNN on synthetic observations (i.e., taken-out model simulations) which provide a ground truth even in the future. Specifically, each DNN pretrained on the remaining 21 CMIP6 simulations is fine-tuned on the left-out simulation for the corresponding scenario by updating its weights on the (simulated) historical data from 1850 to 2022—which represent the training set during this phase (*SI Appendix*, Fig. S1*B*). In other words, the DNNs that were initially pretrained to reproduce the CMIP6 models are now fine-tuned on the historical data simulated by the left-out model, and the same CO_2_ equivalent values of the pretraining phase are used as input. The 21 fine-tuned DNNs are then used to project global surface air temperature maps from 2023 to 2098 (test set) to reproduce the temperatures projected by the left-out model in the long-term future. This procedure is then repeated across the 22 CMIP6 models and the three SSP scenarios, thus providing multiple validation points and testing combinations.

The goal of the leave-one-out cross-validation described above and applied to simulation data is to test the capacity of the proposed TL approach before applying the same method to real observational data and constraining the warming projections, which is done in the next step. Indeed, as was done for the leave-one-out cross-validation, one DNN is pretrained for each CMIP6 model to map the CO_2_ equivalent values previously described to the corresponding surface air temperature global maps from 1850 to 2098 for the three SSP scenarios. This results in the implementation and pretraining of a total of 66 DNNs (*SI Appendix*, Fig. S1*A*). Then, using an ensemble technique (93) (to address epistemic uncertainty) and the TL strategy, the DNNs weights and biases are fine-tuned 5 times independently on the historical BEST dataset (1979 to 2016, 2021, 2022; training set), each time perturbed through the addition of a noise randomly sampled from a Gaussian distribution (thus addressing aleatoric uncertainty) (*SI Appendix*, Fig. S1*C*). The years 2017 to 2020 are reserved for testing purposes during this phase, as the hyperparameters are the same as the DNNs used in the leave-one-out cross-validation except for the learning rate (see *DNNs* for further details).

### DNNs.

The DNNs designed and implemented for each model and scenario share the same architecture and hyperparameters configuration.

Four deconvolutional (or transposed convolutional) layers (98) are used to generate temperature maps from CO_2_ equivalent scalar values. The scalar input is fed to a dense layer made up of 4 × 8 × 128 neurons. Then, the four deconvolutional layers have the role of modeling the correlated spatial information and upsampling it to perform the deconvolutions and reach the spatial resolution of the target map. Specifically, each deconvolutional layer is characterized by 128 kernels with size 10 × 10 and stride equal to 2. This configuration allows the spatial dimensions of the activation volume received by the layer as input to be doubled. The last deconvolutional layer returns an activation volume of size 64 × 128 × 128. A final convolutional layer with a single kernel of size 5 × 5 and stride equal to 1 is needed to refine the spatial information generated by the previous deconvolutional layers and generate the final near-surface air temperature map of size 64 × 128.

The best set of hyperparameters was found after a trial-and-error procedure involving several configurations. We tested different learning rates for the pretraining by progressively increasing the value from 1e-8 to 1e-2. We selected a learning rate equal to 1e-4 as it revealed a good trade-off between generalization accuracy and convergence time, even across different hyperparameter configurations. In the end, the Adam optimizer (99), a learning rate of 1e-4, a batch size of 8, and 500 epochs were used for the pretraining.

During TL, we fine-tuned the pretrained layers selecting a lower learning rate to not dramatically change the values of the weights adjusted during the pretraining. This is usually done when training on new data with the aim of keeping the old knowledge previously acquired and transferring it to the new learning (100). We found good performance with a learning rate about an order of magnitude smaller than the one used during the pretraining, which is a common practice in fine-tuning. We used the same hyperparameters for leave-one-out cross-validation and fine-tuning on observations phases, except for the learning rate. Indeed, with the aim of taking into account the lower number of observational data available for fine-tuning (1979 to 2022)—~4 times less than those available during the leave-one-out cross-validation (1850–2022)—we utilized a learning rate equal to 0.25e-5 during the leave-one-out cross-validation and equal to 1e-5 during the fine-tuning on observational data. The higher learning rate with a lower number of training data helped to reduce the risk of overfitting.

The strategy of freezing some layers during TL was tested as well, but it led to worse results. The final set of hyperparameters for TL is Adam optimizer (99), a batch size of 16, 500 epochs, and learning rate equal to 0.25e-5 for leave-one-out cross-validation and 1e-5 for TL on observational data.

The DNN architecture is the same for both training and TL phases. The loss function is a standard mean absolute error and both the annual CO_2_ equivalent values and the surface air temperature maps are scaled using Min-Max Normalization in the 0 to 1 range. Leaky Rectified Linear Unit activation function (101) was selected for the hidden layers and a sigmoid was used for the output layer because of the 0 to 1 range of Min-Max Normalization of both input and output.

### Metrics.

Temperature anomalies (also referred to as “warming values”) are computed at the grid-point level for both CMIP6 projections and DNNs predictions, each relative to its own climatology. Following Ribes et al. (37), smoothing splines with 20 degrees of freedom are also applied at the grid-point level to CMIP6 projections to reduce the contribution of internal variability. The number of degrees of freedom was tuned according to our data to balance the smoothness and fit of the resulting time series. In order to compute the spatial averages of the maps predicted by the DNNs and simulated by CMIP6 models, a latitude-weighted spatial average is employed. The weights scale each point according to the area it represents depending on the specific latitude.

In each iteration of the leave-one-out cross-validation, in which a CMIP6 model is removed from the ensemble (referred to as taken-out model *i*), the following metrics are computed, with results summarized in *SI Appendix*, Table S2.

$$DNNs\;ensemble\;per\;year={{\mathrm{DE}}_{i}}^{\left(y\right)}=\frac{1}{21}\sum_{m\;=\;1}^{21}{\hat{T}}_{m,i}^{\left(y\right)}$$

where:

• y∈{2081,...,2098}
• m∈{1,...,21} is the index of one of the 21 remaining CMIP6 models that are fine-tuned on the taken-out model *i*
• $i\in \left\{1,...,22\right\}$ is the index of one of the 22 CMIP6 models that is removed from the ensemble during the leave-one-out cross-validation
• ${\hat{T}}_{m,i}^{\left(y\right)}$ is the global average warming value (baseline: 1850 to 1900) predicted by the DNN (pretrained on the $m_{\mathrm{th}}$ CMIP6 model and fine-tuned on the taken-out model *i*) for the year y

$$Global\;average\;{error}_{i}=\frac{1}{18}\sum_{y\;=\;2081}^{2098}\left({{\mathrm{DE}}_{i}}^{\left(y\right)}-T_{i}^{\left(y\right)}\right)$$

where:

$T_{i}^{\left(y\right)}$ is the global average warming value (baseline: 1850 to 1900) simulated by the CMIP6 taken-out model *i* for the year y

$$Global\;{RMSE}_{i}=\sqrt{\frac{\sum_{y=2081}^{2098}{\left({{\mathrm{DE}}_{i}}^{\left(y\right)}-T_{i}^{\left(y\right)}\right)}^{2}}{18}}$$

$$median\;per\;year\left(21\;DNNs\;ensemble\right)={\mathrm{med}}_{DNNs,i}^{\left(y\right)}=median\;of\left\{{\hat{T}}_{m,i}^{\left(y\right)}\right\}$$

$$5\%\;per\;year\left(21\;DNNs\;ensemble\right)={5\%}_{DNNs,i}^{\left(y\right)}=5\%\;of\;\left\{{\hat{T}}_{m,i}^{\left(y\right)}\right\}$$

$$95\%\;per\;year\left(21\;DNNs\;ensemble\right)={95\%}_{DNNs,i}^{\left(y\right)}=95\%\;of\;\left\{{\hat{T}}_{m,i}^{\left(y\right)}\right\}$$

$$5\%\;per\;year\left(21\;CMIP6\;ensemble\right)={5\%}_{CMIP6,i}^{\left(y\right)}=5\%\;of\;\left\{T_{m,i}^{\left(y\right)}\right\}$$

$$95\%\;per\;year\left(21\;CMIP6\;ensemble\right)=95{\%}_{CMIP6,i}^{\left(y\right)}=95\%\;of\;\left\{T_{m,i}^{\left(y\right)}\right\},$$

where:

• $\left\{{\hat{T}}_{m,i}^{\left(y\right)}\right\}$ is the set of global average warming values (baseline: 1850 to 1900) predicted by the 21 DNNs (each pretrained on CMIP6 model m and fine-tuned on the taken-out model *i*) for the year y
• $\left\{T_{m,i}^{\left(y\right)}\right\}$ is the set of global average warming values (baseline: 1850 to 1900) simulated by CMIP6 model m when model *i* is used as taken-out model

$$Avg\;{\mathrm{med}}_{DNNs,i}=\frac{1}{18}\sum_{y\;=\;2081}^{2098}\left({\mathrm{med}\;}_{DNNs,i}^{\left(y\right)}\right)$$

$$Avg\;{5\%}_{DNNs,i}=\frac{1}{18}\sum_{y\;=\;2081}^{2098}\left({5\%}_{DNNs,i}^{\left(y\right)}\right)$$

$$Avg\;{95\%}_{DNNs,i}=\frac{1}{18}\sum_{y\;=\;2081}^{2098}\left({95\%}_{DNNs,i}^{\left(y\right)}\right)$$

$$Avg\;{5\%}_{CMIP6,i}=\frac{1}{18}\sum_{y\;=\;2081}^{2098}\left({5\%}_{CMIP6,i}^{\left(y\right)}\right)$$

$$Avg\;{95\%}_{CMIP6,i}=\frac{1}{18}\sum_{y\;=\;2081}^{2098}\left({95\%}_{CMIP6,i}^{\left(y\right)}\right)$$

$$\%Uncertainty\;{reduction}_{i}=\frac{\left(Avg\;95{\%}_{CMIP6,i}\;-\;Avg\;5{\%}_{CMIP6,i}\right)-\left(Avg\;95{\%}_{DNNs,i}\;-\;Avg\;5{\%}_{DNNs,i}\right)}{\left(Avg\;95{\%}_{CMIP6,i}\;-\;Avg\;5{\%}_{CMIP6,i}\right)}*100$$

$${Avg}_{i}=\frac{1}{18}\sum_{y\;=\;2081}^{2098}\left(T_{i}^{\left(y\right)}\right)$$

$${Accuracy}_{i}=Avg\;{\mathrm{med}}_{DNNs,i}-Avg_{i}$$

The mean values reported at the bottom of *SI Appendix*, Table S2 are computed as follows:

$$Mean\;global\;average\;error=\frac{1}{22}\sum_{i=1}^{22}\left|{\mathrm{Global average error}}_{i}\right|$$

$$Mean\;global\;RMSE=\frac{1}{22}\sum_{i=1}^{22}\left({\mathrm{Global RMSE}}_{i}\right)$$

$$Mean\;accuracy=\frac{1}{22}\sum_{i=1}^{22}\left({\mathrm{Accuracy}}_{i}\right)$$

Some of these values are used to plot *SI Appendix*, Figs. S5 and S6. Indeed, the following quantities are used to plot the light blue and dark blue bars corresponding to each iteration of the leave-one-out cross-validation, in which CMIP6 model *i* is removed from the ensemble and used as taken-out model. Specifically, *Avg 5%_CMIP6,i_* and *Avg 95%_CMIP6,i_* are the average 5% and average 95% of the global temperatures simulated by the remaining CMIP6 models of the ensemble and used to plot the light blue bar. *Avg med_CMIP6,i_* is the median of the global temperatures simulated by the remaining CMIP6 models and used to plot the corresponding red line.

*Avg 5%_DNNs,i_* and *Avg 95%_DNNs,i_* are the average 5% and average 95% of the global temperatures predicted by the DNNs (fine-tuned on taken-out model i) in each iteration and used to plot the dark blue bar. *Avg med_DNNs_* is the median of the global temperatures predicted by the DNNs (fine-tuned on taken-out model i) in each iteration and used to plot the corresponding white line.

*Avg_i_* is the average global temperature simulated by the taken-out CMIP6 model *i* and used to plot the black and dashed lines.

## Supplementary Material

Appendix 01 (PDF)

Movie S1. Results of the leave-one-out cross-validation approach (here for FGOALS-f3-L) when the training set is increased from 1850–1900 to 1850–2022. Global average warming (baseline: 1850–1900) projected by the DNNs ensemble (average across DNNs; bold blue line) for each SSP scenario and FGOALS-f3-L simulation data (bold red line). The projections are generated after TL each DNN on the FGOALS-f3-L historical simulations. Pink shadings show the training sets. The 5 to 95% ranges are reported for the DNNs (dark blue shading; numerical values for the 5 to 95% range of warming prediction in 2098 are present in square brackets), the smoothed CMIP6 simulations (light blue shading), and the original CMIP6 simulations (dashed gray lines). Available at: https://github.com/francescoimmorlano/transferring-climate-change-physical-knowledge

## Data Availability

The datasets used in this study are freely accessible, with any restriction, from the following public repositories: CMIP6 data (https://doi.org/10.24381/cds.c866074c) (102), BEST data: https://berkeleyearth.org/data/ (90), The source code, along with Fig. 1, *SI Appendix*, Fig. S4 and S7 for all the iterations of the leave-one-out cross-validation, and Movie S1 are available on GitHub at https://github.com/francescoimmorlano/transferring-climate-change-physical-knowledge (103). The data and the results are archived on Zenodo at the following DOI: https://doi.org/10.5281/zenodo.15001003 (104).
