# Supplementary material for: Transferring climate change physical knowledge
Source: Proc Natl Acad Sci U S A. 2025 Apr 8;122(15):e2413503122. doi: 10.1073/pnas.2413503122 (PMC12012549; doi:10.1073/pnas.2413503122)
Supplement: Supplementary file 1 — Appendix 01 (PDF) [file pnas.2413503122.sapp.pdf]

PNAS

Supporting Information for
Transferring climate change physical knowledge

Francesco Immorlano[1,2,3*], Veronika Eyring[4,5], Thomas le Monnier de Gouville[6,7], Gabriele Accarino[1,3,6], Donatello Elia[1], Stephan Mandt[2,3], Giovanni Aloisio[1,8], Pierre Gentine[3,6]

[1] Centro Euro-Mediterraneo sui Cambiamenti Climatici (CMCC) Foundation — Euro-Mediterranean Center on Climate Change, Lecce 73100, Italy
[2] Department of Computer Science, University of California, Irvine, CA 92697
[3] Learning the Earth with Artificial Intelligence and Physics, New York, NY 10027
[4] Department of Earth System Model Evaluation and Analysis, Deutsches Zentrum für Luft- und Raumfahrt e.V., Institut für Physik der Atmosphäre, Oberpfaffenhofen, Weßling 82234 Germany
[5] Department of Climate Modelling, University of Bremen, Institute of Environmental Physics, Bremen 28359, Germany
[6] Department of Earth and Environmental Engineering, Columbia University, New York, NY 10027
[7] Ecole Polytechnique, Palaiseau 91120, France
[8] Department of Engineering for Innovation, University of Salento, Lecce 73100, Italy

*Corresponding author: Francesco Immorlano
Email: francesco.immorlano@cmcc.it

This PDF file includes:

Supporting Information Text
Figures S1 to S13
Tables S1 to S8
Movie S1
SI References

Supporting Information Text

Additional results. In the present supplementary section, we provide additional insights and results to clarify further and support the interpretation of our approach.

Pre-training

Fig. S2 shows the predictions generated by the 22 Deep Neural Networks (DNNs), each trained on a different Coupled Model Intercomparison Project Phase 6 (CMIP6) simulation for each Shared Socioeconomic Pathway (SSP). These DNNs are pretrained on the full 1850–2098 period (validation years: 2070–2080) to map the annual CO_2 equivalent scalar value to the corresponding global surface temperature field for the same year (Fig. S1A). The DNNs accurately replicate the CMIP6 models' simulations they are trained on while showing smoother, less variable predictions. This is because they are designed to capture the forced climate response, not interannual variability (as CO_2 equivalent is the sole input variable). This confirms that the DNNs effectively capture both historical and future temporal patterns during the pretraining, aligning closely with the CMIP6 climate signals in response to the CO_2 equivalent forcing.

To prove that also the spatial information is retrieved by the DNNs during this phase, Fig. S3 shows—for each SSP—the difference of the DNNs ensemble with respect to the CMIP6 ensemble averaged over the years 2070–2080 used as validation during the pretraining phase. The DNNs can reproduce the temperature maps without any sign of overfitting, even on the data that was reserved for validation.

Leave-one-out cross-validation

One of the main contributions of the present work is the generation of global surface air temperature maps instead of just scalar values. This allows taking into account the pattern effect which is critical for climate sensitivity. As shown in Fig. S4, the 21 pretrained DNNs (which emulate the CMIP6 models they were trained on—Fig. S4A–C) exhibit regional differences with respect to the taken-out model (MIROC6 in Fig. S4). These differences are then adjusted by fine-tuning the pretrained DNNs over the historical period (1850–2022) of the taken-out model simulation, treated as "synthetic observation" (MIROC6 in Fig. S4). We note a substantial reduction of the regional differences exhibited by the DNNs, confirming that the pattern effect has been considered (Fig. S4D–F).

The achieved results prove that the proposed Transfer Learning (TL) approach is able to adjust the pretrained DNNs to the temporal (Fig. 1) and regional patterns (Fig. S4) simulated by the taken-out model even in the long-term future. To this extent, the years reserved for training (1850–2022) during the leave-one-out cross-validation are critical to allow the DNNs to capture the climate change signals from the taken-out model simulations. Movie S1 reveals that those signals appear evident from 2000–2015 and are exploited by the DNNs to produce increasingly accurate and precise projections up to the end of the century.

To give a complete representation of the leave-one-out cross-validation results, together with information about the position of each taken-out model's projection within the ensemble range, Fig. S5 is provided. This figure compares the confidence range of the temperatures projected by the CMIP6 models and the DNNs for each taken-out model (as described in the section Metrics of Materials and Methods). It can be evinced that the proposed approach proves effective in narrowing the temperature uncertainty range (i.e., increasing the precision), even for those taken-out models that belong to the extremes of the 5–95% range, thus for low- and high-sensitivity climate models (e.g., IITM-ESM and UKESM-1-0-LL across all SSPs).

Concerning the accuracy of the long-term temperature projections, the leave-one-out cross-validation does not exhibit a uniform behavior across the taken-out models and SSPs. In very few cases, the taken-out simulations are overestimated or underestimated, while a very good accuracy is achieved in others. When the DNNs after TL miss the projection from the taken-out model, they are still much closer and in the right direction compared to the pretrained DNNs (before TL). We believe that this is an expected behavior as the only information available to the DNNs to project temperatures close to the taken-out model projections is the knowledge gathered during the pretraining phase (on the entire 1850–2098 period), which is then combined with the information of the temperatures simulated by the taken-out models in 1850–2022 (acquired during the TL). We also note that we are only using one initial condition ensemble member. This inherently results in

some uncertainties, which might explain the variability in the skill across the different SSPs. We also point out that the taken-out models that do not work well tend to have a very abrupt response after 1990, and we have a very simple treatment of aerosol as input for the DNNs, which could also explain some of the discrepancies. It should also be noted that these results in Fig. S5 are computed for the temperatures projected by the DNNs in 2081–2098, which is the period in the future that exhibits the highest uncertainty and is more difficult to predict (1).
To further show the capacity of our method to replicate CMIP6 models simulations even without any model family lineage, the leave-one-out cross-validation was also performed by excluding, at each iteration, the taken-out model as well as those sharing the same atmospheric component. Specifically, the "atmospheric family membership" was first identified based on the atmospheric component of each CMIP6 model reported in Table S1. Afterward, the leave-one-out cross-validation was performed by taking out the selected model, along with its family members. For instance, when ACCESS-CM2 was taken out, KACE-1-0-G and UKESM1-0-LL were excluded as well. The results remain approximately the same whether the family members are excluded (Fig. S6) or not (Fig. S5). This confirms that our proposed TL approach is robust and not biased by specific models that share the same lineage.
In addition, the ability of the proposed TL approach to effectively reduce the uncertainty of the CMIP6 simulations is further demonstrated by performing a time reversal test (Fig. S7). In particular, the 2023–2098 time period was used as training set and 1850–2022 as test set in the leave-one-out cross-validation procedure. As can be noticed in Fig. S7, when ACCESS-CM2 was taken out, the DNNs were able to successfully reverse time across model cases as the DNNs predictions show a reduced 5–95% range compared to CMIP6 simulations, even if there are uncertainties in the historical aerosol forcing which make this experiment challenging.

Transfer Learning on observations
The results just discussed represent a proof of the effectiveness before applying TL on observational data to reduce models' uncertainty in future temperature projections. Furthermore, the projections generated by the DNNs ensemble after TL on observational data were compared to those produced by DNNs trained solely on observations (Fig. S8). While the latter can replicate historical temperature data accurately, they are inherently limited to past and present climate patterns without the added knowledge of projected future trends derived from CMIP6 simulations. Consequently, due to the absence of this future climate "knowledge", the observation-only DNNs produce future temperature projections that deviate significantly from expected trends and align poorly with CMIP6 models, leading to unrealistic and unlikely results. This highlights the advantage of our TL approach, where the pretrained DNNs integrate the comprehensive insights from future CMIP6 projections with observed temperature trends, ultimately generating more plausible future projections.
It is also worth noting that in Fig. 2 the DNNs fine-tuned on observational data exhibit a nearly symmetric uncertainty reduction, with both the high and low ends reduced by comparable amounts relative to CMIP6 simulations. This could be due to the observational data used during TL, which serves as a constraint pulling the pretrained DNNs projections closer to observed historical temperature patterns. This constraint applies to both the high and low ends of the DNNs range. Since observations do not contain the range of extremes often seen in individual CMIP6 simulations, both ends of the uncertainty range are effectively "pulled" toward the observed mean response, thus promoting symmetry. Indeed, Fig. S5 and S6 show that the symmetrical uncertainty reduction effect does not hold uniformly across all take-out models when CMIP6 simulations are used as "synthetic observations." Earth system models simulations lack the low variability and smooth trends observed in actual historical data, and they sometimes introduce asymmetrical uncertainty adjustments across models. For instance, such asymmetric uncertainty reductions are shown for taken-out models such as CNRM-ESM2-1 and MRI-ESM2-0 in SSP2-4.5. In these cases, although the taken-out model simulation is mostly located in the central part of the uncertainty range of the remaining CMIP6 simulations, the DNNs reduce the uncertainty following an asymmetrical pattern. This analysis underscores how variability within the CMIP6 simulations can impact the symmetry of uncertainty reduction when using taken-out models in leave-one-out cross-validation instead of real observational constraints.

Comparison with state-of-the-art bias correction methods. Several methods have been introduced in the scientific literature to reduce the bias of global climate models (2–4). In particular, Wu et al. (2) investigate the potential of four bias correction (BC) techniques for narrowing the uncertainty in temperature and precipitation over the globe and individual continents. The BC methods are Delta Change (DC) (5), Quantile Mapping (QM) (6, 7), Nonstationary Cumulative-Distribution Function-matching (CNCDFm) (8), and Bias Correction and Spatial Disaggregation (BCSD) (9–11). The authors quantify the uncertainty in temperature and precipitation projections arising from the outputs of 21 CMIP5 and 26 CMIP6 models in 1955–2099 under SSPs 1-2.6, 2-4.5, and 5-8.5 (for CMIP6). They apply the decomposition of the uncertainty into three sources—model uncertainty, scenario uncertainty, and internal variability—following the method developed by Hawkins and Sutton (12, 13). The methodology adopted by the authors is thoroughly described in (2).

We applied the same uncertainty partitioning on both the 22 unconstrained CMIP6 simulations used in our work and the predictions made by the DNNs after fine-tuning on the observational data. We did not compute the 10-year running average on the temperatures projected by the DNNs after TL on observational data since they predict the forced temperature response exhibiting less variability than CMIP6 simulations. The period chosen to calculate the multi-year average temperature baseline is 1979–1999 since 1979 is the first year for which observational data are available in this work. The choice of the baseline period is not a concern as past studies have demonstrated that it has almost no effect on the uncertainty decomposition results (14). This allowed us to evaluate the model uncertainty, internal variability, and scenario uncertainty and compute both the total (Fig. S11) and fractional uncertainty (Fig. S12). We also assessed the reductions of model uncertainty in the near term (2030–2039; Table S7) and long term (our work: 2085–2094; BCSD, QM, DC, CNCDFm: 2090–2099; Table S8) and compared them to the reductions obtained with the BC methods (2). Concerning the long term, the period selected is 2085–2094 as the 10-year running mean (applied on CMIP6 simulations from 1850 to 2098) removes the first five and the last four years—thus, the last year available is 2094. The authors focus on the percentage reduction of model uncertainty for the four BC methods as they find that the reduction of the total uncertainty is primarily due to a reduction in model uncertainty. Tables S7 and S8 show that our TL approach reaches a greater percentage reduction in both long- and near-term model uncertainty compared to all the BC methods. We also computed the percentage reduction of the fractional model uncertainty, which is equal to 76.6% in the near term and 46.5% in the long term in our case (see Figs. S12 and S13).

Additional results. Fig. S4 and S7 for all the taken-out models are available at https://github.com/francescoimmorlano/transferring-climate-change-physical-knowledge (45).

Supplementary Figures and Tables

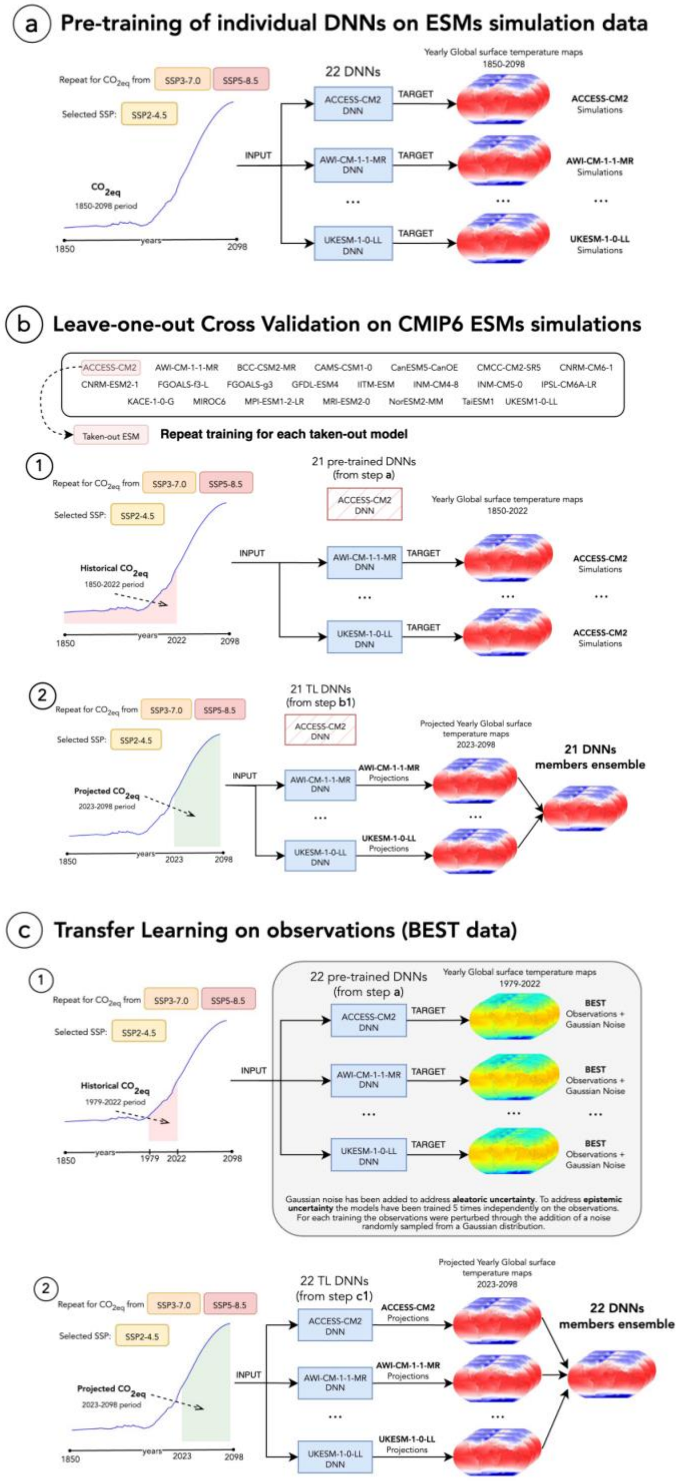

Figure S1. Leave-one-out cross-validation and TL workflows.

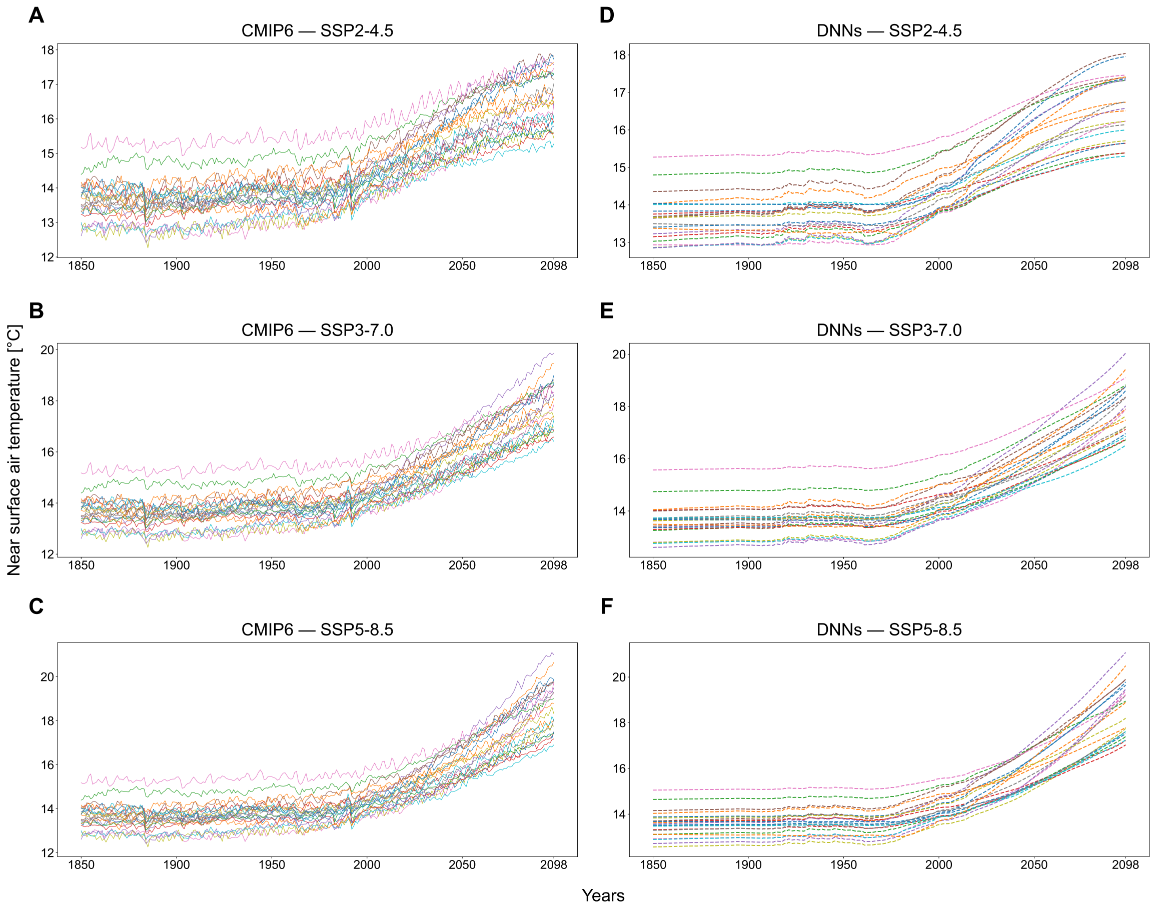

Figure S2. Pretraining results. (A–F) Comparison between CMIP6 simulations (A–C, thin dotted lines) and global mean temperatures generated by the pretrained DNNs (D–F, thin dotted lines). The temperatures are generated after pretraining each DNN on one of the CMIP6 simulations (the one with the same color) from 1850 to 2098, reserving the years 2070–2080 for validation.

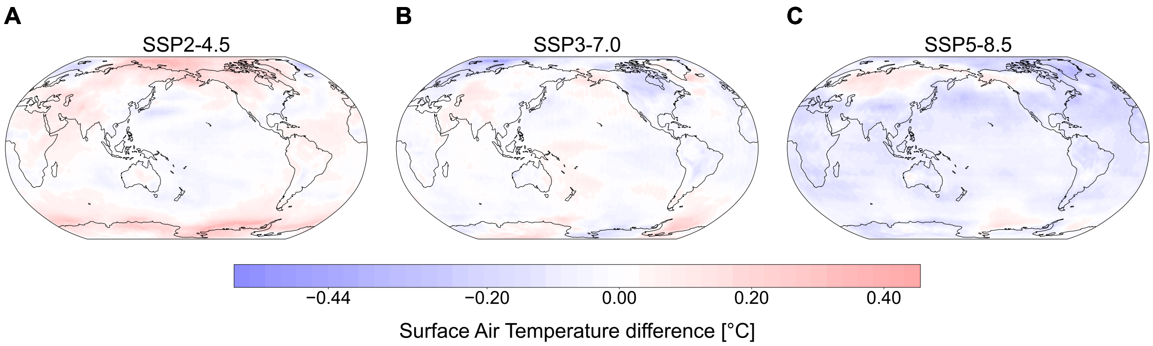

Figure S3. Pretraining results on validation years (2070–2080). (A–C) Average surface air temperature difference maps generated by the DNNs ensemble with respect to the CMIP6 ensemble for each SSP. Each DNN was trained on one of the CMIP6 simulations from 1850 to 2098, reserving the years 2070–2080 for validation. Then, the DNNs predictions, as well as CMIP6 simulations, were averaged over the years 2070–2080.

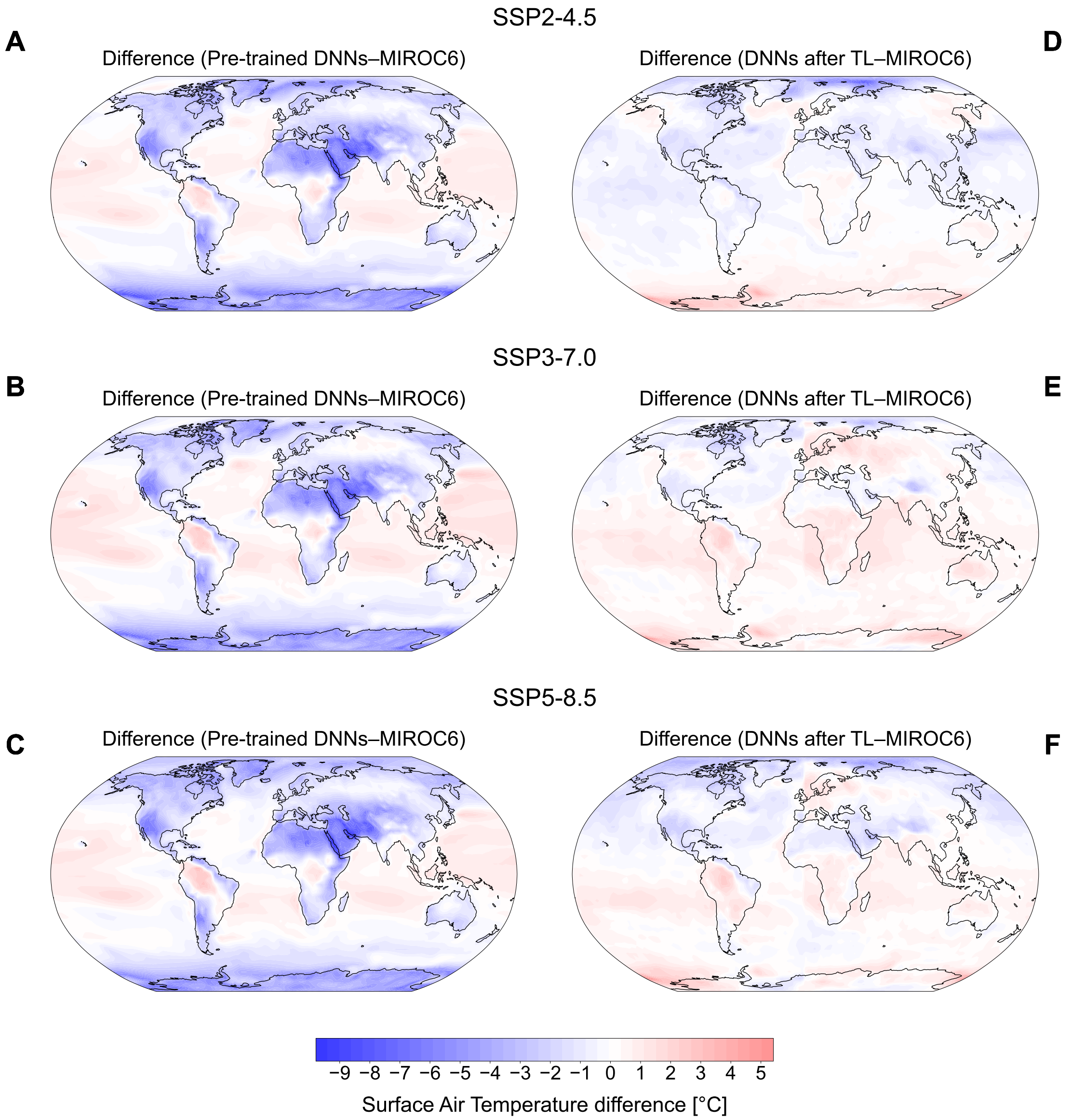

Figure S4. Comparison of the results of pretrained DNNs and DNNs transfer learned on "synthetic observations" (here for MIROC6). (A–F) Average surface air temperature difference maps generated by the DNNs with respect to the MIROC6 simulation for each SSP in the 2081–2098 period. The maps were generated after pretraining 21 DNNs, each on a different CMIP6 simulation (excluding MIROC6), from 1850 to 2098 (A–C) and after transfer learning the pretrained DNNs on MIROC6 historical simulation data (1850–2022) (D–F). Then, the DNNs ensemble predictions were averaged in 2081–2098.

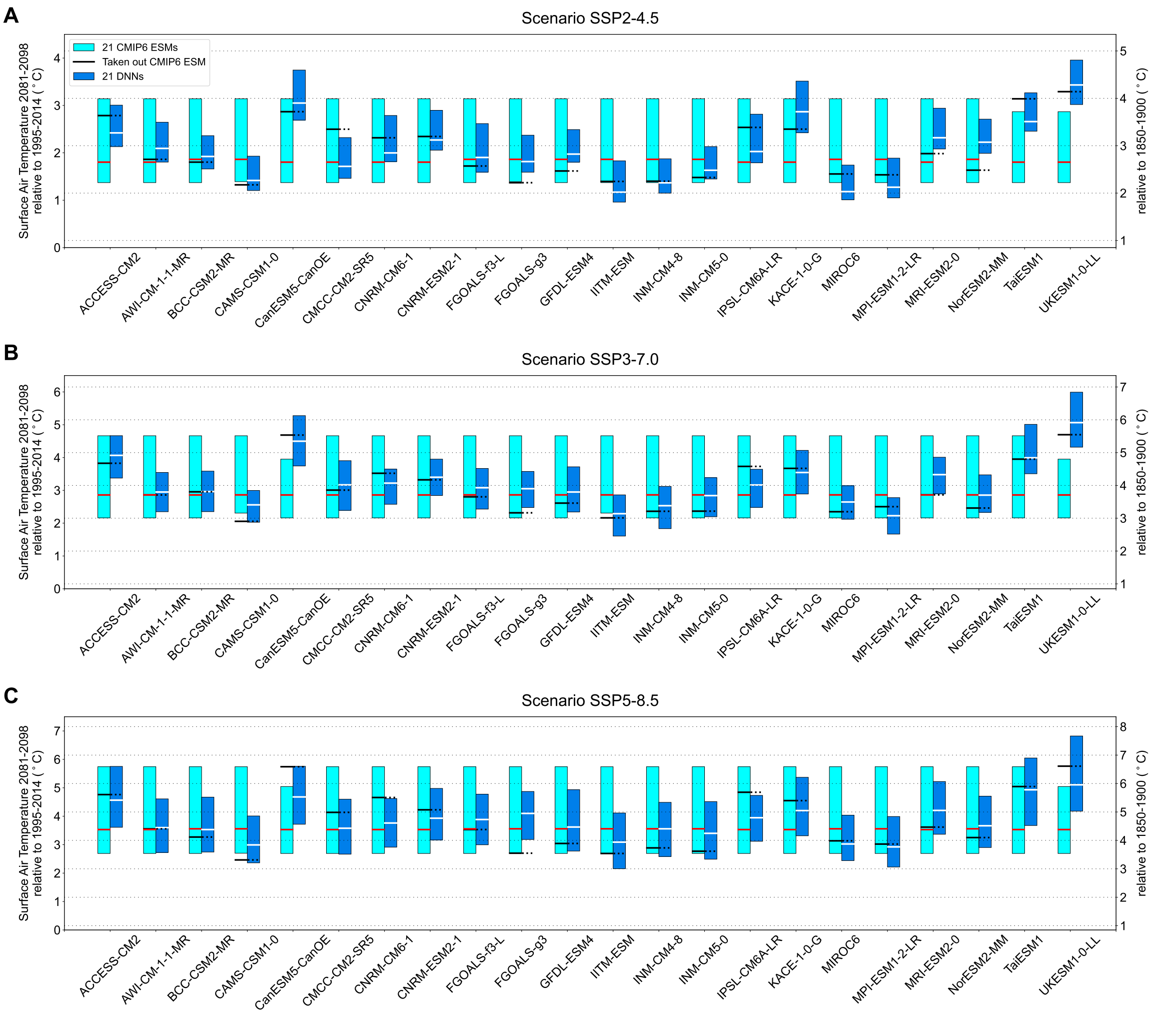

Figure S5. Leave-one-out cross-validation results. (A–C) Accuracy and precision of the DNNs after each iteration of the leave-one-out cross-validation procedure. Each panel shows the global average 5–95% warming ranges for the long-term period (2081–2098)—relative to 1995–2014 (left y axis) and 1850–1900 (right y axis)—simulated by the remaining 21 CMIP6 models (light blue bars) and predicted by the 21 DNNs ensemble after TL (dark blue bars). The temporally averaged median values of CMIP6 simulations (red line) and DNNs predictions (white line) are also reported. Black lines represent the temporal average of the global temperature simulated by the taken-out CMIP6 model. The results are produced for SSPs 2-4.5 (A), 3-7.0 (B), and 5-8.5 (C).

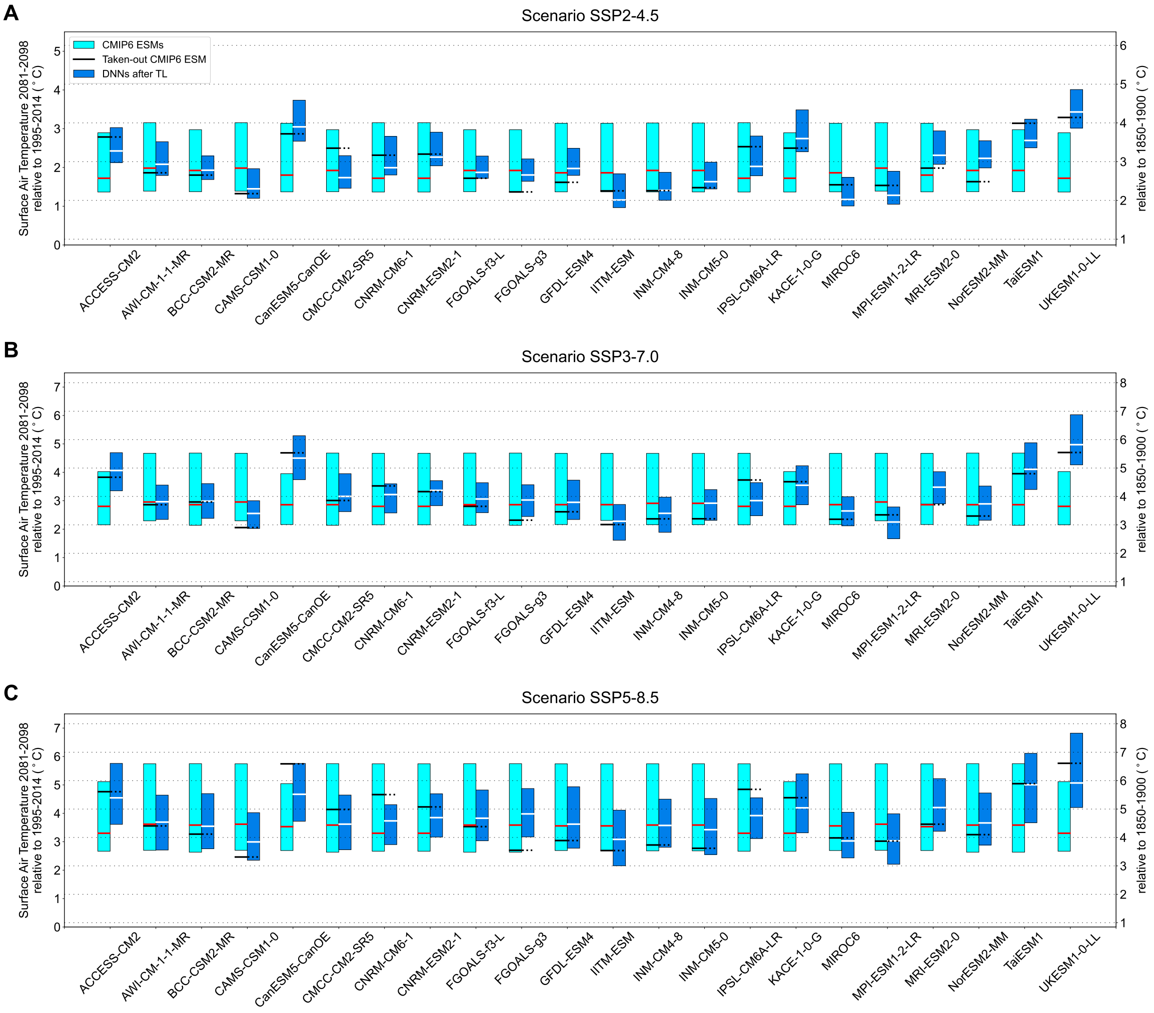

Figure S6. Leave-one-out cross-validation results excluding (in turn) the taken-out CMIP6 model and those sharing its atmospheric model. (A–C) Accuracy and precision of the DNNs after each iteration of the leave-one-out cross-validation procedure. Besides the taken-out CMIP6 model, in each iteration we also excluded the other models sharing the same atmospheric component (Table S1). Each panel shows the global average 5–95% warming ranges for the long-term period (2081–2098)—relative to 1995–2014 (left y axis) and 1850–1900 (right y axis)—simulated by the remaining CMIP6 models (light blue bars) and predicted by the DNNs ensemble after TL (dark blue bars). The temporally averaged median values of CMIP6 simulations (red line) and DNNs predictions (white line) are also reported. Black lines represent the temporal average of the global temperature simulated by the taken-out CMIP6 model. The results are produced for SSPs 2-4.5 (A), 3-7.0 (B), and 5-8.5 (C).

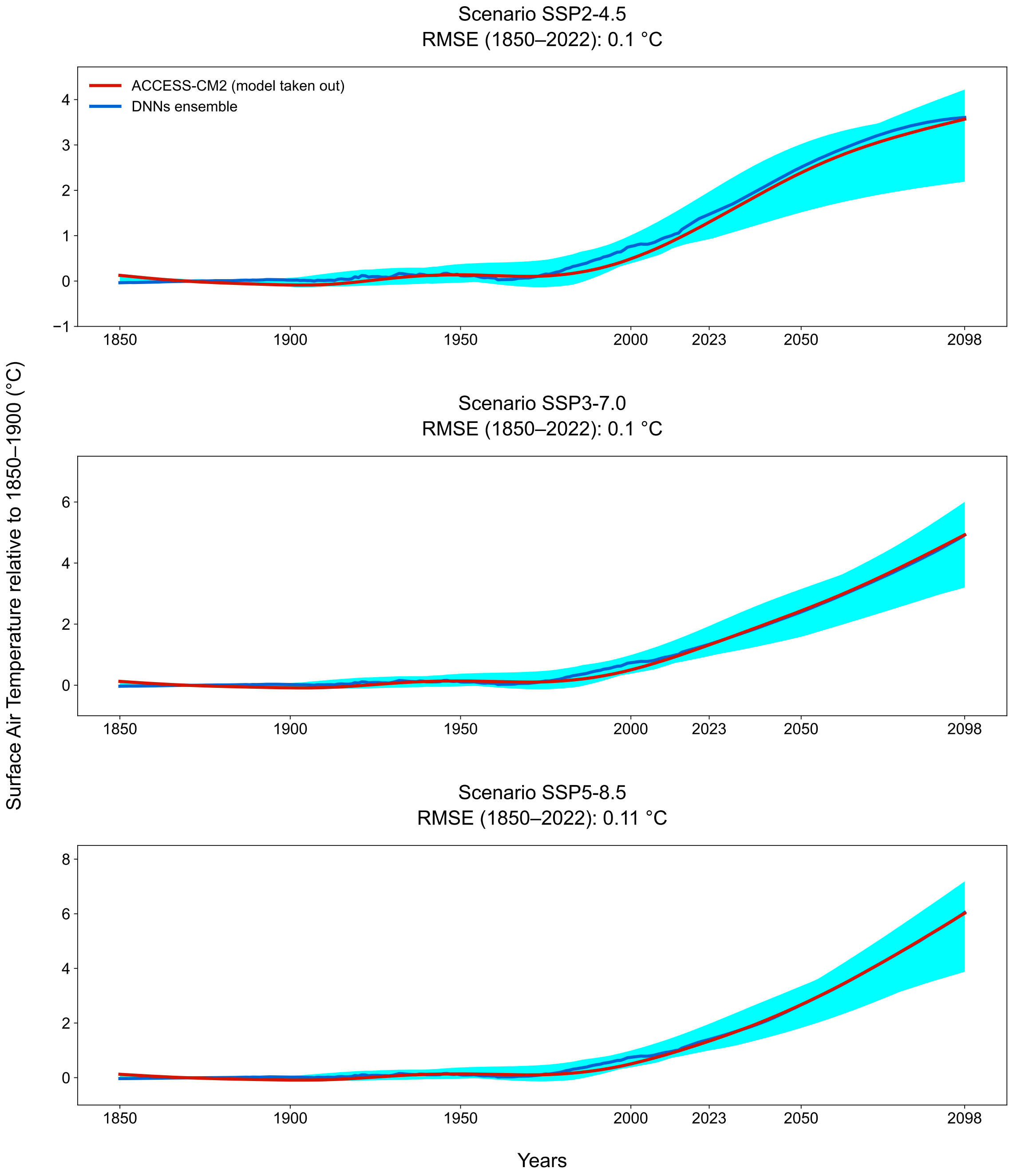

Figure S7. Leave-one-out cross-validation (reversing time, here for ACCESS-CM2). Global average warming (baseline: 1850–1900) projected by the DNNs ensemble (average across DNNs; bold blue line) for each scenario and ACCESS-CM2 smoothed simulation data (bold red line). The historical temperatures in 1850–2022 are generated after transfer learning each DNN on the ACCESS-CM2 projections (2023–2098). Pink shadings show the training set (2023–2098); dark blue shadings show the 5–95% range of the DNNs ensemble; light blue shadings show the 5–95% range of the smoothed CMIP6 ensemble.

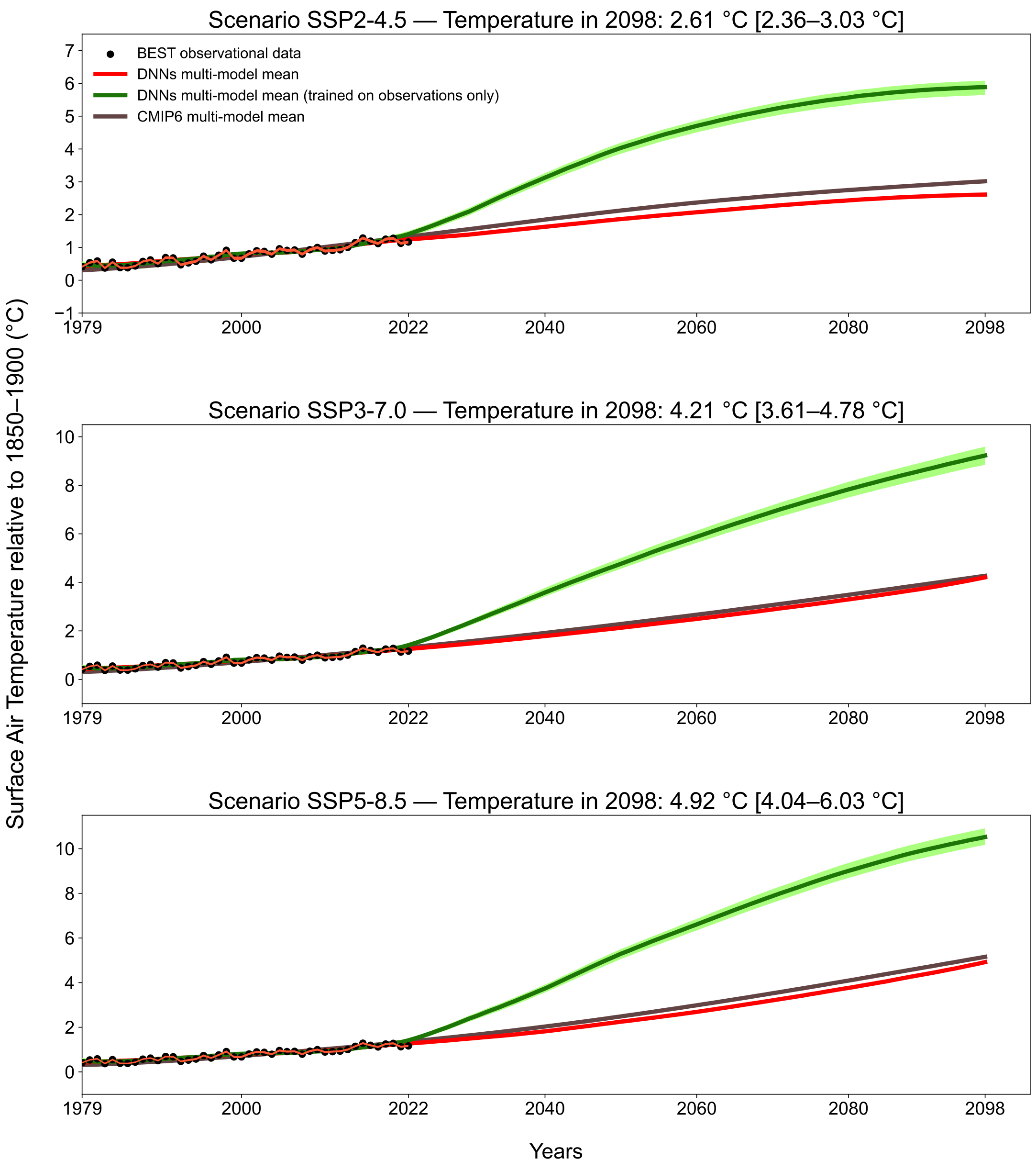

Figure S8. Comparison of DNNs trained on observations only (without CMIP6 model pretraining) and DNNs pretrained on CMIP6 models and then transfer learned on observational data. DNNs ensemble (average across DNNs) projections of global average warming relative to 1850–1900 for each SSP scenario. The bold red line refers to the ensemble projections of DNNs transfer learned (training set, pink shading: 1979–2016, 2021, 2022; validation set, grey shading: 2017–2020) on BEST-perturbed historical observational data (black dots). The bold green line refers to the ensemble projections of DNNs solely trained on the aforementioned BEST dataset. The 5–95% ranges of DNNs transfer learned on observations (light red shading), DNNs trained on observations only (light green shadings), and smoothed unconstrained CMIP6 (light brown shading) are also reported. The smoothed, unconstrained CMIP6 ensemble (bold brown line) is shown as well. For each plot, numerical values of the 5–95% range for temperature prediction in 2098 (the last projection year) are present in square brackets.

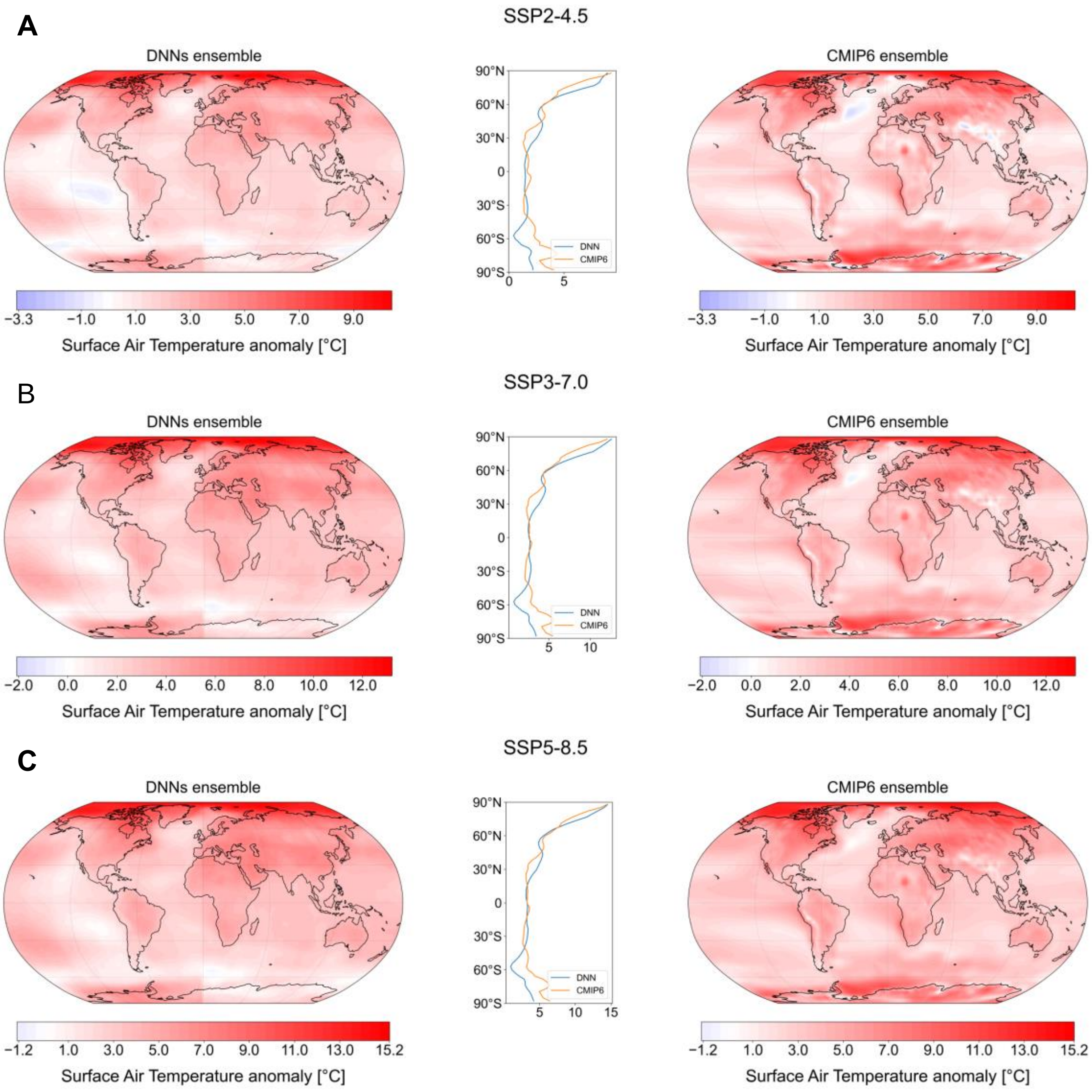

Figure S9. Long-term surface air temperature anomaly maps and temperature variation across latitudes. (A–C) Surface air temperature anomaly maps in 2081–2098 relative to 1980–1990. They are computed by averaging over time the temperature maps generated by the DNNs (left) and CMIP6 models (right) for SSP2-4.5 (A), SSP3-7.0 (B), and SSP5-8.5 (C). The maps are produced by the DNNs after transfer learning them on observations. The variation of temperatures across latitudes is also reported (center).

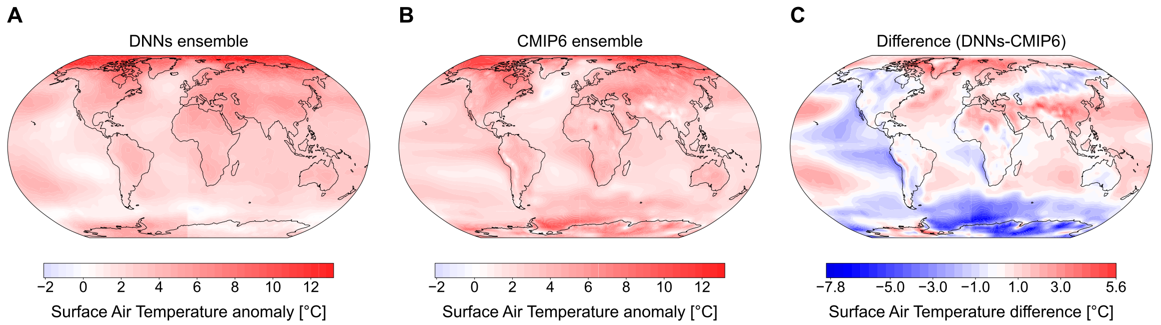

Figure S10. Long-term surface air temperature anomaly maps. (A–C) Surface air temperature anomaly maps in 2081–2098 relative to 1980–1990 for SSP3-7.0. They are computed by averaging in time (between 2081 and 2098) the temperature maps generated by the DNNs (A) and CMIP6 models (B). The difference between DNNs and CMIP6 average maps is also reported (C). The maps are produced by the DNNs after transfer learning them on observations.

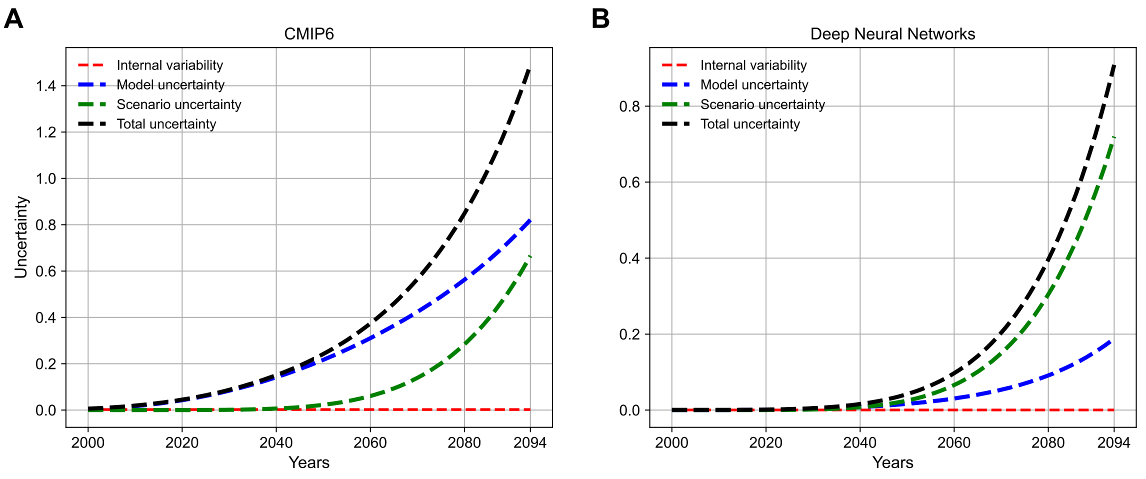

Figure S11. Uncertainty components. Uncertainty components (i.e., total uncertainty, model uncertainty, scenario uncertainty, and internal variability) of unconstrained CMIP6 simulations (A) and predictions made by the DNNs after TL on observational data (B) computed in 2000–2094.

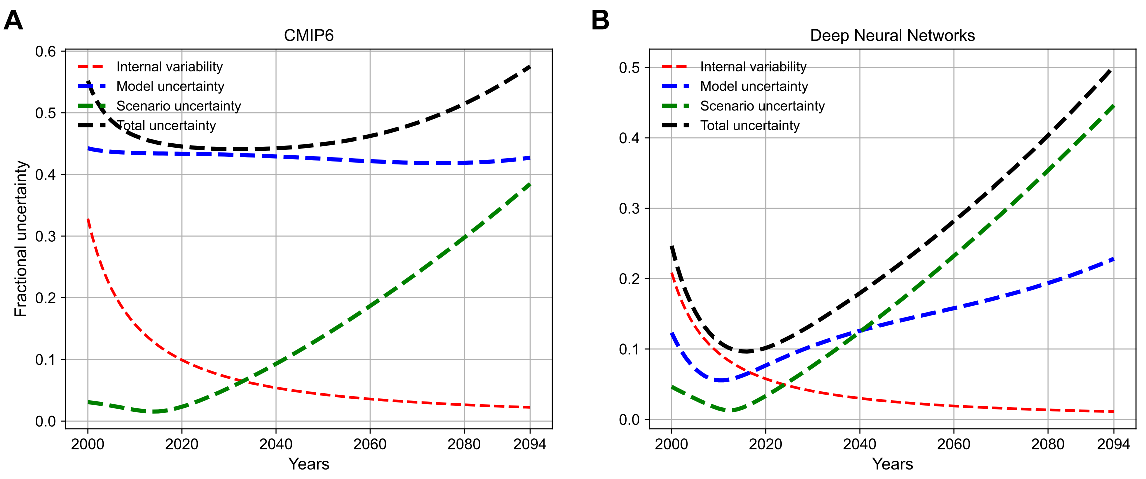

Figure S12. Fractional uncertainty components. Fractional uncertainty components (i.e., total uncertainty, model uncertainty, scenario uncertainty, and internal variability) of unconstrained CMIP6 simulations (A) and predictions made by the DNNs after TL on observational data (B) computed in 2000–2094.

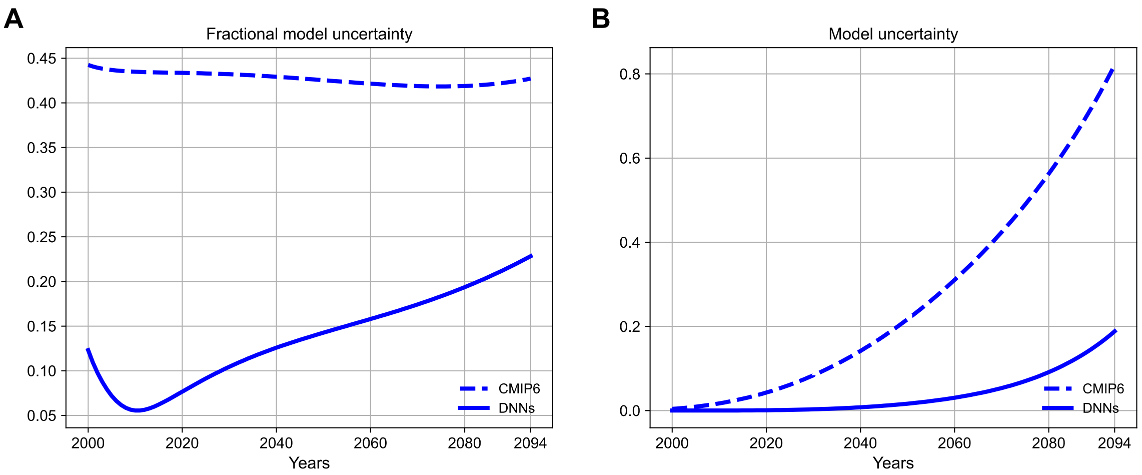

Figure S13. Model uncertainty components. Fractional model uncertainty (A) and model uncertainty (B) components of unconstrained CMIP6 and predictions made by the DNNs after TL on observational data, computed in 2000–2094.

Movie S1. Results of the leave-one-out cross-validation approach (here for FGOALS-f3-L) when the training set is increased from 1850–1900 to 1850–2022. Global average warming (baseline: 1850–1900) projected by the DNNs ensemble (average across DNNs; bold blue line) for each SSP scenario and FGOALS-f3-L simulation data (bold red line). The projections are generated after TL each DNN on the FGOALS-f3-L historical simulations. Pink shadings show the training sets. The 5 to 95% ranges are reported for the DNNs (dark blue shading; numerical values for the 5 to 95% range of warming prediction in 2098 are present in square brackets), the smoothed CMIP6 simulations (light blue shading), and the original CMIP6 simulations (dashed gray lines). Available at: https://github.com/francescoimmorlano/transferring-climate-change-physical-knowledge (45).

Table S1. CMIP6 Earth system models. List of CMIP6 models used in this work, their atmospheric model lineage, and ensemble member. Notice that CMCC-CM2-SR5, NorESM2-MM, and TaiESM1 do not share the same atmospheric model, but are still related. Indeed, they leverage atmospheres based on the Community Atmosphere Model (15) developed at the National Center for Atmospheric Research. Thus, they are considered sharing the same lineage in this work.

**Coupled model**	**Atmospheric model**	**Reference**	**Member**
ACCESS-CM2	MetUM-HadGEM3-GA7.1, 192x144, 85 lv	Bi et al., 2020 (16)	r1i1p1f1
AWI-CM-1-1-MR	ECHAM6.3.04p1, 384x192, T127L95, 95 lv	Semmler et al, 2020 (17)	r1i1p1f1
BCC-CSM2-MR	BCC-AGCM3-MR, 320×160, 46 lv	Wu et al., 2021(18)	r1i1p1f1
CAMS-CSM-1-0	ECHAM5_CAMS, 320x160, 31 lv	Xin-Yao et al., 2019 (19)	r1i1p1f1
CanESM5-CanOE	CanAM5, 128x64, 49 lv	Swart et al., 2019 (20)	r1i1p2f1
CMCC-CM2-SR5	CAM5.3, 288×192, 30 lv	Cherchi et al., 2019 (21)	r1i1p1f1
CNRM-CM6-1	ARPEGE 6.3 256×128, 91 lv, T127 Gr 24572 gb	Voldoire et al., 2019 (22)	r1i1p1f2
CNRM-ESM2-1	ARPEGE 6.3, 720×360, T127 Gr 24572 gb, 91 lv	Séférian et al., 2019 (23)	r1i1p1f2
FGOALS-f3-L	FAMIL2.2, 360x180, 32 lv	He et al., 2019 (24)	r1i1p1f1
FGOALS-g3	GAMIL3, 180×80, hybrid, 26 lv	Pu et al., 2020 (25)	r1i1p1f1
GFDL-ESM4	GFDL-AM4.1, 360x180, 49 lv	Dunne et al., 2020 (26)	r1i1p1f1
IITM-ESM	IITM-GFSv1, 192×94, 64 lv	Krishnan et al., 2021 (27)	r1i1p1f1
INM-CM4-8	INM-AM4-8, 180x120, 21 lv	Volodin et al., 2018 (28)	r1i1p1f1
INM-CM5-0	INM-AM5-0, 180x120, 73 lv	Volodin and Gritsun, 2018 (29)	r1i1p1f1
IPSL-CM6A-LR	LMDZ NPv6, 144×143, N96L79, 79 lv	Boucher et al., 2020 (30)	r1i1p1f1
KACE-1-0-G	MetUM-HadGEM3-GA7.1, 192x144, 85 lv	Lee et al., 2020 (31)	r1i1p1f1
MIROC6	CCSR AGCM, 256×128, T85L81, 81 lv	Tatebe et al., 2019 (32)	r1i1p1f1
MPI-ESM1-2-LR	ECHAM6.3, 192×96, T63L95, 47 lv	Mauritsen et al., 2019 (33)	r1i1p1f1
MRI-ESM2-0	MRI-AGCM3.5, 320×160, TL159L80, 80 lv	Yukimoto et al., 2019 (34)	r1i1p1f1

NorEMS2-MM	CAM-Oslo, 288×192, 32 lv;	Seland et al., 2020 (35)	r1i1p1f1
TaiESM1	TaiAM1, 288×192, 30 lv	Wang et al., 2021 (36)	r1i1p1f1
UKESM1-0-LL	MetUM-HadGEM3-GA7.1, 192x144, 85 lv	Sellar et al., 2019 (37)	r1i1p1f2

Table S2. Leave-one-out cross-validation results. Global average error, global RMSE, percentage of uncertainty reduction, accuracy (of the DNNs ensemble with respect to the taken-out model), average 5% and 95% with respect to the average of temperatures (predicted by the DNNs) in 2081–2098 computed in the leave-one-out cross-validation approach for each of the 22 CMIP6 models and the three SSPs (see Metrics section in Materials and Methods). Each model is the taken-out model considered as synthetic ground truth.

Model	Scenario	Global average error [°C]	Global RMSE [°C]	% uncertainty reduction	Accuracy	Avg 5% [°C]	Avg 95% [°C]
ACCESS-CM2	SSP2-4.5	-0.28	0.28	54.07	-0.37	2.75	3.62
	SSP3-7.0	0.2	0.2	47.56	0.29	4.05	5.34
	SSP5-8.5	-0.05	0.05	26.28	-0.14	4.29	6.42
AWI-CM-1-1-MR	SSP2-4.5	0.19	0.19	53.89	0.16	2.82	3.68
	SSP3-7.0	-0.02	0.07	51.11	0.02	3.35	4.55
	SSP5-8.5	-0.09	0.09	33.83	-0.06	3.71	5.62
BCC-CSM2-MR	SSP2-4.5	0.14	0.14	63.13	0.11	2.31	3.0
	SSP3-7.0	-0.04	0.07	49.97	0.01	3.03	4.25
	SSP5-8.5	0.33	0.36	33.19	0.28	3.41	5.35
CAMS-CSM1-0	SSP2-4.5	0.1	0.1	60.63	0.03	1.81	2.53
	SSP3-7.0	0.37	0.38	60.48	0.41	2.61	3.57
	SSP5-8.5	0.63	0.63	42.49	0.45	2.95	4.6
CanESM5-CanOE	SSP2-4.5	0.16	0.17	34.23	0.1	3.96	4.99
	SSP3-7.0	-0.24	0.25	20.81	-0.26	5.03	6.55
	SSP5-8.5	-1.13	1.13	13.91	-1.14	4.97	7.0
CMCC-CM2-SR5	SSP2-4.5	-0.77	0.77	55.48	-0.86	2.5	3.34
	SSP3-7.0	0.1	0.12	39.72	0.1	3.44	4.92
	SSP5-8.5	-0.65	0.65	33.08	-0.66	3.69	5.63
CNRM-CM6-1	SSP2-4.5	-0.19	0.2	50.68	-0.29	2.7	3.62
	SSP3-7.0	-0.29	0.29	57.21	-0.25	3.44	4.49
	SSP5-8.5	-0.91	0.92	41.11	-0.87	3.78	5.49
CNRM-ESM2-1	SSP2-4.5	0.03	0.1	56.95	-0.06	2.77	3.58
	SSP3-7.0	0.06	0.06	55.42	0.12	3.57	4.66
	SSP5-8.5	-0.15	0.17	38.14	-0.23	3.92	5.71
FGOALS-f3-L	SSP2-4.5	0.18	0.18	48.13	0.09	2.61	3.59
	SSP3-7.0	0.22	0.23	50.1	0.18	3.42	4.65
	SSP5-8.5	0.21	0.22	38.13	0.22	3.98	5.77
FGOALS-g3	SSP2-4.5	0.45	0.45	59.77	0.38	2.5	3.26
	SSP3-7.0	0.62	0.63	56.11	0.66	3.38	4.45
	SSP5-8.5	1.25	1.25	41.61	1.31	4.09	5.78
GFDL-ESM4	SSP2-4.5	0.39	0.39	64.16	0.3	2.38	3.05
	SSP3-7.0	0.3	0.3	44.46	0.27	2.91	4.28
	SSP5-8.5	0.59	0.6	25.88	0.5	3.34	5.49
IITM-ESM	SSP2-4.5	-0.14	0.14	55.29	-0.26	1.8	2.64
	SSP3-7.0	0.03	0.07	49.92	0.07	2.43	3.65
	SSP5-8.5	0.37	0.38	31.63	0.33	2.96	4.94
INM-CM4-8	SSP2-4.5	-0.06	0.06	61.85	-0.14	1.92	2.63
	SSP3-7.0	-0.01	0.07	47.34	0.06	2.58	3.87
	SSP5-8.5	0.54	0.55	34.22	0.54	3.33	5.23
INM-CM5-0	SSP2-4.5	0.11	0.11	64.64	0.04	2.12	2.78
	SSP3-7.0	0.34	0.36	52.82	0.35	2.91	4.07
	SSP5-8.5	0.54	0.55	30.88	0.49	3.18	5.17
IPSL-CM6A-LR	SSP2-4.5	-0.47	0.47	44.73	-0.58	2.8	3.84
	SSP3-7.0	-0.74	0.74	53.07	-0.63	3.49	4.64
	SSP5-8.5	-1.0	1.01	44.01	-0.97	4.14	5.75

KACE-1-0-G	SSP2-4.5	0.35	0.35	43.17	0.33	3.55	4.62
	SSP3-7.0	-0.17	0.18	45.87	-0.14	4.0	5.33
	SSP5-8.5	-0.39	0.39	28.66	-0.37	4.42	6.48
MIROC6	SSP2-4.5	-0.34	0.34	61.89	-0.42	1.57	2.28
	SSP3-7.0	0.23	0.25	58.24	0.26	2.68	3.7
	SSP5-8.5	-0.02	0.03	44.56	-0.15	2.98	4.58
MPI-ESM1-2-LR	SSP2-4.5	-0.21	0.21	57.31	-0.3	1.9	2.7
	SSP3-7.0	-0.38	0.38	54.5	-0.36	2.48	3.6
	SSP5-8.5	-0.12	0.12	39.44	-0.16	3.05	4.8
MRI-ESM2-0	SSP2-4.5	0.38	0.38	55.26	0.3	2.75	3.59
	SSP3-7.0	0.53	0.54	53.9	0.59	3.57	4.7
	SSP5-8.5	0.58	0.59	36.43	0.59	4.08	5.91
NorESM2-MM	SSP2-4.5	0.71	0.71	60.73	0.63	2.52	3.23
	SSP3-7.0	0.48	0.49	53.9	0.41	2.84	3.96
	SSP5-8.5	0.56	0.56	37.91	0.47	3.43	5.23
TaiESM1	SSP2-4.5	-0.36	0.37	57.98	-0.48	2.94	3.73
	SSP3-7.0	0.19	0.22	40.1	0.08	4.04	5.51
	SSP5-8.5	-0.1	0.15	18.72	-0.04	4.23	6.58
UKESM1-0-LL	SSP2-4.5	0.2	0.21	40.85	0.11	3.69	4.62
	SSP3-7.0	0.4	0.41	14.29	0.4	5.05	6.69
	SSP5-8.5	-0.27	0.28	-10.87	-0.6	4.93	7.55
Mean values	SSP2-4.5	0.28	0.29	-	0.29	-	-
	SSP3-7.0	0.27	0.29	-	0.27	-	-
	SSP5-8.5	0.48	0.49	-	0.48	-	-

Table S3. Global 5–95% warming ranges for the long-term period (2081–2100) relative to 1995–2014 for SSPs 2-4.5, 3-7.0, and 5-8.5. We also report the percentage reduction in the spread of our approach with respect to each other 5–95% range for the same scenario. Note that the 5–95% ranges for Ribes et al. and this work are computed in the 2081–2098 time period. The remaining ones are computed in the 2081–2100 time period.

	SSP2-4.5 (°C)	SSP3-7.0 (°C)	SSP5-8.5 (°C)
Ribes et al. (38) (2081–2098)	1.22–2.44 (47%)	2.07–3.47 (35%)	2.4–4.53 (25%)
Liang et al. (39)	1.33–2.72 (53%)	2.28–3.85 (42%)	2.6–4.86 (29%)
Tokarska et al. (40)	1.04–2.56 (57%)	1.75–3.63 (52%)	2.09–4.75 (40%)
IPCC WG1 AR6 (41)	1.2–2.6 (54%)	2.0–3.7 (46%)	2.4–4.8 (33%)
fair-calibrate v1.4.1 (42)	1.06–2.66 (59%)	1.63–3.18 (41%)	2.12–4.37 (15%)
fair-calibrate v1.4.0 (42)	1.06–2.68 (60%)	1.85–3.52 (46%)	2.32–4.78 (35%)
CMIP6 ensemble	1.37–3.13 (63%)	2.17–4.63 (63%)	2.69–5.71 (47%)
This work (2081–2098)	1.45–2.1	2.42–3.33	2.8–4.4

Table S4. Global 5–95% warming ranges for the near- (2021–2040) and mid-term (2041–2060) periods relative to 1995–2014 for SSP2-4.5, 3-7.0 and 5-8.5 scenarios. We also report the percentage reduction in the spread of our approach with respect to each other 5–95% range for the same scenario.

	Time period	SSP2-4.5 (°C)	SSP3-7.0 (°C)	SSP5-8.5 (°C)
IPCC WG1 AR6 (41)	2021–2040	0.4–0.9 (70%)	0.4–0.9 (62%)	0.5–1.0 (52%)
	2041–2060	0.8–1.6 (55%)	0.9–1.7 (59%)	1.1–2.1 (45%)
fair-calibrate v1.4.1 (42)	2021–2040	0.41–0.91 (70%)	0.41–0.89 (60%)	0.45–0.98 (55%)
	2041–2060	0.72–1.57 (58%)	0.79–1.54 (56%)	0.94–1.97 (47%)
fair-calibrate v1.4.0 (42)	2021–2040	0.40–0.86 (67%)	0.41–0.83 (55%)	0.48–1.02 (56%)
	2041–2060	0.71–1.54 (57%)	0.86–1.56 (53%)	1.01–2.06 (48%)
This work	2021–2040	0.49–0.64	0.56–0.75	0.54–0.78
	2041–2060	0.85–1.21	1.15–1.48	1.16–1.71

Table S5. Mean and standard deviation of warming for 2041–2050 and 2091–2100 periods relative to 1850–1900 for SSP2-4.5, 3-7.0 and 5-8.5 scenarios. Note that the values for this work are computed in the 2091–2098 time period.

	Time period	SSP2-4.5	SSP3-7.0	SSP5-8.5
OSCAR v3.1 (43)	2041–2050	1.75 ± 0.17	1.87 ± 0.21	2.04 ± 0.19
	2091–2100	2.50 ± 0.25	3.50 ± 0.32	4.16 ± 0.38
This work	2041–2050	1.76 ± 0.09	1.97 ± 0.1	2.05 ± 0.16
	2091–2098	2.59 ± 0.25	4.0 ± 0.34	4.67 ± 0.6

Table S6. Time to 1.5°C and 2°C thresholds. Years to reach 1.5°C and 2°C thresholds relative to 1850–1900 time period according to SSP2-4.5, 3-7.0, and 5-8.5 scenarios.

	Threshold	SSP2-4.5 (°C)	SSP3-7.0 (°C)	SSP5-8.5 (°C)
Diffenbaugh and Barnes (44)	1.5°C	2033 (2028–2039)	2035 (2030–2040)	-
	2°C	2049 (2043–2055)	2050 (2043–2058)	-
This work	1.5°C	2035 (2031–2040)	2031 (2028–2034)	2030 (2028–2036)
	2°C	2057 (2049–2068)	2047 (2043–2051)	2045 (2040–2051)

Table S7. Model uncertainty reduction in the near term. Percentage reduction of model uncertainty in the near term (2030–2039).

Our work	BCSD	QM	DC	CNCDFm
95.5%	70.5%	70.5%	61.4%	49.5%

Table S8. Model uncertainty reduction in the long term. Percentage reduction of model uncertainty in the long term (our work: 2085–2094; BCSD, QM, DC, CNCDFm: 2090–2099).

This work	BCSD	QM	DC	CNCDFm
79.4%	72.4%	72.2%	52.8%	57.4%

Supporting Information References

1. C. Tebaldi, *et al.*, Climate model projections from the Scenario Model Intercomparison Project (ScenarioMIP) of CMIP6. *Earth System Dynamics* 12, 253–293 (2021).
2. Y. Wu, *et al.*, Quantifying the Uncertainty Sources of Future Climate Projections and Narrowing Uncertainties With Bias Correction Techniques. *Earths Future* 10 (2022).
3. D. Carvalho, S. Cardoso Pereira, A. Rocha, Future surface temperatures over Europe according to CMIP6 climate projections: an analysis with original and bias-corrected data. *Clim Change* 167, 10 (2021).
4. Z. Xu, Y. Han, C.-Y. Tam, Z.-L. Yang, C. Fu, Bias-corrected CMIP6 global dataset for dynamical downscaling of the historical and future climate (1979–2100). *Sci Data* 8, 293 (2021).
5. R. Beyer, M. Krapp, A. Manica, An empirical evaluation of bias correction methods for palaeoclimate simulations. *Climate of the Past* 16, 1493–1508 (2020).
6. D. Maraun, *et al.*, Towards process-informed bias correction of climate change simulations. *Nat Clim Chang* 7, 764–773 (2017).
7. E. P. Maurer, D. W. Pierce, Bias correction can modify climate model simulated precipitation changes without adverse effect on the ensemble mean. *Hydrol Earth Syst Sci* 18, 915–925 (2014).
8. C. Miao, L. Su, Q. Sun, Q. Duan, A nonstationary bias-correction technique to remove bias in GCM simulations. *Journal of Geophysical Research: Atmospheres* 121, 5718–5735 (2016).
9. A. W. Wood, E. P. Maurer, A. Kumar, D. P. Lettenmaier, Long-range experimental hydrologic forecasting for the eastern United States. *Journal of Geophysical Research: Atmospheres* 107 (2002).
10. A. W. Wood, L. R. Leung, V. Sridhar, D. P. Lettenmaier, Hydrologic Implications of Dynamical and Statistical Approaches to Downscaling Climate Model Outputs. *Clim Change* 62, 189–216 (2004).
11. L. Xu, A. Wang, Application of the Bias Correction and Spatial Downscaling Algorithm on the Temperature Extremes From CMIP5 Multimodel Ensembles in China. *Earth and Space Science* 6, 2508–2524 (2019).
12. E. Hawkins, R. Sutton, The potential to narrow uncertainty in projections of regional precipitation change. *Clim Dyn* 37, 407–418 (2011).
13. E. Hawkins, R. Sutton, The Potential to Narrow Uncertainty in Regional Climate Predictions. *Bull Am Meteorol Soc* 90, 1095–1108 (2009).
14. T. Zhou, J. Lu, W. Zhang, Z. Chen, The Sources of Uncertainty in the Projection of Global Land Monsoon Precipitation. *Geophys Res Lett* 47 (2020).
15. R. B. Neale, *et al.*, Description of the NCAR Community Atmosphere Model (CAM 5.0). (2012). https://doi.org/10.5065/wgtk-4g06.
16. D. Bi, *et al.*, Configuration and spin-up of ACCESS-CM2, the new generation Australian Community Climate and Earth System Simulator Coupled Model. *Journal of Southern Hemisphere Earth Systems Science* 70, 225–251 (2020).
17. T. Semmler, *et al.*, Simulations for CMIP6 With the AWI Climate Model AWI-CM-1-1. *J Adv Model Earth Syst* 12, e2019MS002009 (2020).
18. T. Wu, *et al.*, BCC-CSM2-HR: a high-resolution version of the Beijing Climate Center Climate System Model. *Geosci Model Dev* 14, 2977–3006 (2021).
19. R. Xin-Yao, *et al.*, Introduction of CAMS-CSM model and its participation in CMIP6. *Advances in Climate Change Research* 15, 540 (2019).
20. N. C. Swart, *et al.*, The Canadian Earth System Model version 5 (CanESM5.0.3). *Geosci Model Dev* 12, 4823–4873 (2019).
21. A. Cherchi, *et al.*, Global Mean Climate and Main Patterns of Variability in the CMCC-CM2 Coupled Model. *J Adv Model Earth Syst* 11, 185–209 (2019).
22. A. Voldoire, *et al.*, Evaluation of CMIP6 DECK Experiments With CNRM-CM6-1. *J Adv Model Earth Syst* 11, 2177–2213 (2019).
23. R. Séférian, *et al.*, Evaluation of CNRM Earth System Model, CNRM-ESM2-1: Role of Earth System Processes in Present-Day and Future Climate. *J Adv Model Earth Syst* 11, 4182–4227 (2019).

24. B. He, *et al.*, CAS FGOALS-f3-L Model Datasets for CMIP6 Historical Atmospheric Model Intercomparison Project Simulation. *Adv Atmos Sci* 36, 771–778 (2019).
25. Y. Pu, *et al.*, CAS FGOALS-g3 Model Datasets for the CMIP6 Scenario Model Intercomparison Project (ScenarioMIP). *Adv Atmos Sci* 37, 1081–1092 (2020).
26. J. P. Dunne, *et al.*, The GFDL Earth System Model Version 4.1 (GFDL-ESM 4.1): Overall Coupled Model Description and Simulation Characteristics. *J Adv Model Earth Syst* 12, e2019MS002015 (2020).
27. R. Krishnan, *et al.*, The IITM Earth System Model (IITM ESM). [Preprint] (2021). Available at: http://arxiv.org/abs/2101.03410.
28. E. M. Volodin, *et al.*, Simulation of the modern climate using the INM-CM48 climate model. 33, 367–374 (2018).
29. E. Volodin, A. Gritsun, Simulation of observed climate changes in 1850–2014 with climate model INM-CM5. *Earth System Dynamics* 9, 1235–1242 (2018).
30. O. Boucher, *et al.*, Presentation and Evaluation of the IPSL-CM6A-LR Climate Model. *J Adv Model Earth Syst* 12, e2019MS002010 (2020).
31. J. Lee, *et al.*, Evaluation of the Korea Meteorological Administration Advanced Community Earth-System model (K-ACE). *Asia Pac J Atmos Sci* 56, 381–395 (2020).
32. H. Tatebe, *et al.*, Description and basic evaluation of simulated mean state, internal variability, and climate sensitivity in MIROC6. *Geosci Model Dev* 12, 2727–2765 (2019).
33. T. Mauritsen, *et al.*, Developments in the MPI-M Earth System Model version 1.2 (MPI-ESM1.2) and Its Response to Increasing CO2. *J Adv Model Earth Syst* 11, 998–1038 (2019).
34. S. Yukimoto, *et al.*, The Meteorological Research Institute Earth System Model Version 2.0, MRI-ESM2.0: Description and Basic Evaluation of the Physical Component. *Journal of the Meteorological Society of Japan. Ser. II* 97, 931–965 (2019).
35. Ø. Seland, *et al.*, Overview of the Norwegian Earth System Model (NorESM2) and key climate response of CMIP6 DECK, historical, and scenario simulations. *Geosci Model Dev* 13, 6165–6200 (2020).
36. Y.-C. Wang, *et al.*, Performance of the Taiwan Earth System Model in Simulating Climate Variability Compared With Observations and CMIP6 Model Simulations. *J Adv Model Earth Syst* 13, e2020MS002353 (2021).
37. A. A. Sellar, *et al.*, UKESM1: Description and Evaluation of the U.K. Earth System Model. *J Adv Model Earth Syst* 11, 4513–4558 (2019).
38. A. Ribes, S. Qasmi, N. P. Gillett, Making climate projections conditional on historical observations. *Sci Adv* 7, eabc0671 (2021).
39. Y. Liang, N. P. Gillett, A. H. Monahan, Climate Model Projections of 21st Century Global Warming Constrained Using the Observed Warming Trend. *Geophys Res Lett* 47, e2019GL086757 (2020).
40. K. B. Tokarska, *et al.*, Past warming trend constrains future warming in CMIP6 models. *Sci Adv* 6, eaaz9549 (2020).
41. Intergovernmental Panel on Climate Change (IPCC), "Future Global Climate: Scenario-based Projections and Near-term Information" in *Climate Change 2021 – The Physical Science Basis: Working Group I Contribution to the Sixth Assessment Report of the Intergovernmental Panel on Climate Change*, I. P. on C. C. IPCC, Ed. (Cambridge University Press, 2023), pp. 553–672.
42. C. Smith, *et al.*, fair-calibrate v1.4.1: calibration, constraining and validation of the FaIR simple climate model for reliable future climate projections. [Preprint] (2024). Available at: https://egusphere.copernicus.org/preprints/2024/egusphere-2024-708/ [Accessed 27 October 2024].
43. Y. Quilcaille, T. Gasser, P. Ciais, O. Boucher, CMIP6 simulations with the compact Earth system model OSCAR v3.1. *Geosci Model Dev* 16, 1129–1161 (2023).
44. N. S. Diffenbaugh, E. A. Barnes, Data-driven predictions of the time remaining until critical global warming thresholds are reached. *Proceedings of the National Academy of Sciences* 120, e2207183120 (2023).

45. F. Immorlano, Source code from “Transferring climate change physical knowledge” Github. https://github.com/francescoimmorlano/transferring-climate-change-physical-knowledge. Accessed on 25 March 2025.
